# Supplementary material for: Non-pharmacological therapies for postviral syndromes, including Long COVID: a systematic review and meta-analysis protocol
Source: BMJ Open. 2022 Apr 10;12(4):e057885. doi: 10.1136/bmjopen-2021-057885 (PMC9002258; doi:10.1136/bmjopen-2021-057885)
Supplement: Author's manuscript [file bmjopen-2021-057885.draft_revisions.pdf]

BMJ Open is committed to open peer review. As part of this commitment we make the peer review history of every article we publish publicly available.

When an article is published we post the peer reviewers' comments and the authors' responses online. We also post the versions of the paper that were used during peer review. These are the versions that the peer review comments apply to.

The versions of the paper that follow are the versions that were submitted during the peer review process. They are not the versions of record or the final published versions. They should not be cited or distributed as the published version of this manuscript.

BMJ Open is an open access journal and the full, final, typeset and author-corrected version of record of the manuscript is available on our site with no access controls, subscription charges or pay-per-view fees (http://bmjopen.bmj.com).

If you have any questions on BMJ Open's open peer review process please email info.bmjopen@bmj.com

BMJ Open

Post-viral syndromes: a systematic review of symptoms, health impacts, treatments and their implications for the management of Long COVID

Journal:	*BMJ Open*
Manuscript ID	bmjopen-2021-057885
Article Type:	Protocol
Date Submitted by the Author:	30-Sep-2021
Complete List of Authors:	Chandan, Joht; University of Birmingham College of Medical and Dental Sciences<br>Brown, Kirsty; University of Birmingham College of Medical and Dental Sciences<br>Simms-Williams, Nikita; University of Birmingham,<br>Camaradou, Jenny; COVID-END<br>Bashir, Nasir; University of Birmingham<br>Heining, Dominic; University Hospitals Birmingham NHS Foundation Trust<br>Aiyegbusi, Olalekan Lee; University of Birmingham, Centre for Patient-Reported Outcomes Research; University of Birmingham, NIHR Birmingham Biomedical Research Centre, University of Birmingham and University Hospitals Birmingham NHS Foundation Trust<br>Turner, Grace; University of Birmingham, Centre for Patient Reported Outcomes Research<br>Nirantharakumar, Krishnarajah; University of Birmingham, Institute of Applied Health Research<br>Sivan, Manoj; University of Leeds,<br>Khunti, Kamlesh; University of Leicester, Department of Health Sciences<br>Raindi, Devan; University of Birmingham<br>Marwaha, Steven; University of Birmingham College of Life and Environmental Sciences<br>Hughes, Sarah; University of Birmingham College of Medical and Dental Sciences<br>McMullan, Christel; University of Birmingham, Institute of Applied Health Research<br>Calvert , Melanie ; University of Birmingham, Centre for Patient Reported Outcomes Research and Institute of Applied Health Research<br>Haroon, Shamil; University of Birmingham,
Keywords:	COVID-19, REHABILITATION MEDICINE, INFECTIOUS DISEASES

SCHOLARONE™
Manuscripts

For peer review only - http://bmjopen.bmj.com/site/about/guidelines.xhtml

Post-viral syndromes: a systematic review of symptoms, health impacts, treatments and their implications for the management of Long COVID

Running title: Post-viral syndromes and Long COVID

Authors: Joht Singh Chandan[1]*, Kirsty Brown[1]*, Nikita Simms-Williams[1], Jenny Camaradou[2], Nasir Bashir[3], Dominic Heining[4], Olalaken Lee Aiyegbusi[1,5-8], Grace Turner[1,5], Krishnarajah Niratharakumar[1,12], Manoj Sivam[9], Kamlesh Khunti[10], Devan Raindi[3], Steven Marwaha[11], Sarah E. Hughes[1,5,6], Christel McMullan[1,5], Melanie Calvert[1,5-8,13]†, Shamil Haroon[1]†

*Joint first author
†Joint senior author

Corresponding author:
Dr Joht Singh Chandan
Institute of Applied Health Research, University of Birmingham, Birmingham, UK
Joht.chandan@nhs.net

Affiliations:
[1]Institute of Applied Health Research, University of Birmingham, Birmingham, UK
[2]COVID END Evidence Network
[3]School of Dentistry, University of Birmingham, Birmingham, UK
[4] University Hospitals Birmingham NHS Foundation Trust, Birmingham, UK
[5] Centre for Patient Reported Outcomes Research, Institute of Applied Health Research, University of Birmingham, Birmingham, UK
[6]National Institute for Health Research (NIHR) Applied Research Centre West Midlands, Birmingham, UK
[7]Birmingham Health Partners Centre for Regulatory Science and Innovation, University of Birmingham, Birmingham, UK
[8]NIHR Birmingham Biomedical Research Centre, NIHR Surgical Reconstruction and Microbiology Research Centre, University of Birmingham, Birmingham, UK
[9]School of Medicine, University of Leeds, Leeds, UK
[10]Diabetes Research Centre, University of Leicester, Leicester, UK
[11]Institute for Mental Health, University of Birmingham, Birmingham, UK
[12]Midlands Health Data Research UK, Birmingham, UK
[13]Health Data Research UK, London, UK

For peer review only

Word count: 2335

Conflict of interest: There are no conflicts of interest among the authors relevant to this study.

Author statement: The research questions were conceived by JSC, KN, MC and SH. The first draft of the protocol was done by KB, NSW, JSC and SH. Further revisions to the protocol were made by all remaining co-authors (CM, JC, NB, DM, OL, GT, KN, MS, KK, DR, SM, SEH, KM, JL, DW). The final copy of the manuscript was approved by all authors.

Funding and acknowledgments: This work is independent research jointly funded by the National Institute for Health Research (NIHR) and UK Research and Innovation (UKRI) (Therapies for Long COVID in non-hospitalised individuals: From symptoms, patient reported outcomes and immunology to targeted therapies (The TLC Study), COV-LT-0013). The views expressed in this publication are those of the author(s) and not necessarily those of the National Institute for Health Research or the Department of Health and Social Care.

Data sharing: This protocol does not have any data.

Ethics approval: This study does not require ethical approval as it is a secondary analysis of published data.

Transparency statement: The lead author (the manuscript's guarantor) affirms that this manuscript is an honest, accurate, and transparent account of the study being reported; that no important aspects of the study have been omitted; and that any discrepancies from the study as planned (and, if relevant, registered) have been explained.

peer review only

Abstract

Introduction: Post-viral syndromes (PVS) describe the sustained presence of symptoms following an acute viral infection, for months or even years. Exposure to the SARS-CoV-2 virus and subsequent development of COVID-19 has shown to have similar effects with individuals continuing to exhibit symptoms for greater than twelve weeks. The sustained presence of symptoms is variably referred to as 'post COVID-19 syndrome', 'post-COVID condition' or more commonly 'Long COVID'. Knowledge of the long-term health impacts, and treatments for Long COVID are evolving. This review aims to summarise the symptoms, health impacts and treatments for existing post-viral syndromes, to derive evidence relevant to the management of Long COVID.

Methods and analysis: Primary electronic searches will be performed in bibliographic databases including: Embase, MEDLINE, PyscINFO, CINAHL, Cochrane Library, COVID-NMA and preprint servers from 1st January 2001 to inception. At least two independent reviewers will screen each study for inclusion and data will be extracted from all eligible studies onto a data extraction form. The quality of all included studies will be assessed using AMSTAR-2, Cochrane risk of bias tools and the Newcastle-Ottawa grading system. The prevalence of symptoms and health impacts (clinical complications and impacts on quality of life and work capability) for different post-viral syndromes will be narratively described and then pooled using a proportion random-effects meta-analysis where there is sufficient methodological homogeneity. The findings will be narratively compared to existing literature on the symptoms and health impacts of Long COVID. Non-pharmacological treatments for post-viral syndromes, including Long COVID, will be narratively summarised and effect estimates will be pooled using random effects meta-analysis where there is sufficient methodological homogeneity.

Ethics and dissemination: This systematic review does not require ethical approval. The findings from this study will be submitted for peer-reviewed publication, shared at conference presentations, and disseminated to both clinical and patient groups.

Keywords: Post-viral syndromes, COVID-19, Long COVID, rehabilitation, systematic review

For peer review only

Strengths and Limitations of this study

- This review will provide the first comprehensive and systematic summary of the known long-term symptoms and health impacts of previous post-viral syndromes. It will also comprehensively summarise the evidence on treatments for previous post-viral syndromes as well as the treatments for Long COVID.
- This review proposal has been developed with the support of patients and the public, through discussions and patient and public involvement meetings.
- The results of this review will aid clinicians in identifying similarities between known post-viral syndromes and Long COVID and will inform the clinical management of Long COVID and prioritisation of treatments for further evaluation.
- A limitation of the review relates to the heterogeneity in the definition of post-viral syndromes, adding complexity to the synthesis of available evidence across the included syndromes and applicability to Long COVID.

For peer review only

Background

Globally there have been over 223 million cases of COVID-19 with over 4 million associated deaths.[1] The pandemic has triggered a concerted global effort to develop and deliver safe and efficacious vaccinations at record speed, which have now significantly reduced morbidity, mortality, and disease transmission associated with Severe Acute Respiratory Syndrome Coronavirus-2 (SARS CoV-2). [2–4]

Despite such positivity, the long-term sequalae of even mild infections of COVID-19 appear to carry a substantive burden on some patients, their families and health services. Early evidence suggested that the average recovery time following an acute COVID-19 infection is 2-3 weeks, depending on symptom severity.[5,6] However, as the pandemic has progressed it became apparent that not all patients immediately recovered from the initial infection and between 2.3-37% develop symptoms lasting greater than 12 weeks, some of which may be relapsing and remitting.[7,8] However, prevalence estimates in these longer-term symptoms vary because of differences in disease definition (including differences in symptom severity and included symptoms), population surveyed and study design.[8–13] The sustained presence of such symptoms is variably referred to as 'post COVID-19 syndrome' in the UK, 'post-COVID condition' by the World Health Organization or more commonly known as 'Long COVID' (the term used throughout this review) among patient groups.

The prevalence of persistent signs and symptoms, the remitting, relapsing and interchangeable nature of these symptoms, the pathophysiology and clinical course of Long COVID, and the lived experiences of patients, is yet to be fully described. However, many clinical sequalae including, but not limited to, fatigue, respiratory, neurological, psychological and gastroenterological complications that present in patients with Long COVID also feature in a similar pattern following other acute viral infections.[13,14]

An improved understanding of the sequalae and management of existing post-viral syndromes secondary to acute infections may provide further insight and guidance as to the optimal management of patients with Long COVID. Despite the novelty of the condition, it is clear that optimal management involves a multidisciplinary approach and rehabilitation, with patient involvement being a central component.[15,16]

Post-viral syndromes may occur secondary to exposure to a number of acute viral infections, including but not limited to Coronaviridae, Filoviridae, Herpesviridae, Orthomyxoviridae, Picornaviridae, Parvoviridae, Togaviridae, and Flaviviridae, as defined by the International Committee on Taxonomy of Viruses.[17–25] Like Long COVID, the signs and symptoms comprising these post-viral syndromes may include fatigue, musculoskeletal pain (e.g., joint pain), and mood (e.g. feeling depressed) and neurocognitive disturbances (e.g., difficulties with concentration and sleep).[14,26]

Although the pathophysiology of post-viral syndromes is poorly understood, it has been hypothesised that their development is mediated by altered cytokine responses, neuroinflammation, mitochondrial dysfunction and microbiome dysbiosis. [27–31] A culmination of the pathophysiological changes and phenotypes following acute viral

For peer review only - http://bmjopen.bmj.com/site/about/guidelines.xhtml

infections, including COVID-19, have been likened to other poorly understood conditions such as myalgic encephalomyelitis (ME)/chronic fatigue syndrome (CFS).[32] Despite the limited evidence and clinical consensus, guidelines for the management of Long COVID have been developed and trials are underway exploring the impact of pharmaceutical, dietary and lifestyle interventions.[33,34]

Considering the significant individual and societal burden arising from the COVID-19 pandemic there is an urgent need to identify whether the existing knowledge base on similar post-viral syndromes can be applied to support the clinical assessment and management of patients with Long COVID. A comprehensive synthesis of the evidence on post-viral syndromes, including on their therapies, is needed to better support those with Long COVID as well as those with previous post-viral syndromes.

Objectives

This systematic review will summarise the symptoms and health impacts (clinical complications and impacts on quality of life and work capability) of previous post-viral syndromes that follow acute infections. The review will also summarise evidence on non-pharmacological treatments for previous post-viral syndromes and Long COVID. The findings will be used to make recommendations on non-pharmacological treatments for Long COVID as well as previous post-viral syndromes. We have focused on non-pharmacological treatments as pharmacological therapies are currently already reviewed by the UK COVID-19 Therapeutics Panel (UK-CTAP).[35]

This systematic review will be conducted and reported in accordance with the PRISMA guidelines, and this protocol has been written in accordance with the PRISMA-P checklist (Supplementary File 1).[36,37] The review will adhere to this protocol which has also been registered with PROSPERO.

Methods

Eligibility criteria

We will include primary research studies and secondary analyses including systematic reviews, randomised and non-randomised controlled trials, retrospective and prospective observational studies (cohort and case-control studies), cross-sectional studies, and case series. For non-pharmacological treatments effects, we will consider observational study data as well as trial evidence. There will be no restrictions on setting (i.e., community or hospital based). Studies published in the previous 20 years will be included (from 31st December 2001 to present), as this would encompass research on the first SARS and MERS outbreak which are the two pandemic viruses most related to SARS-CoV-2. No language restrictions will be applied. Peer-reviewed published studies, preprint articles and other grey literature will also be included in this review.

We will include studies with a population consisting of adults and/or children with post-viral syndromes persisting for more than 12 weeks. Studies investigating post-viral syndromes

For peer review only

following acute infections from the following families (grouped by the International Committee on Taxonomy of Viruses)[17] will be included in this review. Viruses known to cause symptomatic acute viral infections will be included. This is based on an assessment of clinically relevant viruses extracted from "Mandell, Douglas, and Bennett's Principles and Practice of Infectious Disease, 9th Edition.[38]" Included in brackets are the relevant clinical infections used to inform search terms:

Coronaviridae (Middle East Respiratory Syndrome-Coronavirus, Severe Acute Respiratory Syndrome-Coronavirus, and other coronaviruses), Filoviridae (Ebola, Marburg), Herpesviridae (Epstein-Barr Virus, Cytomegalovirus, Varicella Zoster Virus, Other Human Herpes Viruses, Herpes Simplex Virus), Orthomyxoviridae (Influenza, A, B, C and D), Picornaviridae (Coxsackie, Hepatitis A, Enterovirus including Rhinovirus), Parvoviridae (Parvovirus), Togaviridae (Ross River virus, Chikungunya, other Semliki Forest Complex Viruses), Flaviviridae (West Nile River Virus, Dengue, Yellow Fever, Zika), Reoviridiae (Rotavirus), Paramyxoviridae (Mumps, Measles, Parainfluenza, Respiratory Syncytial Virus, Human Metapneumovirus) Adenoviridiae (Adenoviruses), Caliciviridae (Norovirus, Sapovirus), Astroviridae (Astroviruses). Additionally, when examining treatment options for Long COVID we will also include studies relating to SARS-CoV-2.

Extremely rare viral infections will not be included, as it is thought that these are unlikely to be published research relevant to this review. Predominantly asymptomatic viruses, viruses identified in the last 3 years, and viruses predominantly existing as zoonoses will all be excluded in addition to studies on chronic viral infections including Hepatitis B, Hepatitis C and HIV post-viral syndromes. Studies which include demonstrable reactivation of latent infection (e.g EBV, CMV with detectable viral load) will be excluded. We will exclude *in vitro* and animal studies. Studies that principally focus on ME and CFS without reference to pre-specified viruses will be excluded from the review.

The outcomes considered for this review will be symptoms, health impacts (clinical complications and impacts on quality of life and work capability), and treatments.

Information sources

Primary electronic searches will be performed in Embase, MEDLINE, PyscINFO, CINAHL, COVID-NMA, preprint servers (including Medrxiv and SSRN) and the Cochrane Library from 1st January 2001 to inception. The reference lists of all eligible articles will also be searched to identify any additional eligible studies which were not identified during the original search. Secondary searches will be conducted for grey literature on databases such as Open Grey and Google Scholar (limited to the first 1000 references). Abstracts, conference, and symposia proceedings from relevant organisations relating to virology will be identified and examined.

Search methods for identification of studies

The search strategy broadly includes terms such as: "MERS-COV", "SARS-COV", "Coronavirus", "Ebola", "Epstein-Barr Virus", "EBV", "Cytomegalovirus", "CMV", "Herpes",

For peer review only

"Influenza", "Swine flu", "Coxsackie", "Parvovirus", "Ross River virus", "Chikungunya", "West Nile River virus", "long COVID", "post-acute-COVID", "Dengue", "symptoms", "treatment", "management", "health impact", "complications", "Post-viral" , "quality of life" , "outcomes". A more extensive list of search terms is attached in Supplementary File 2.

Study records

Data management, selection, and data collection processes

The study selection procedure will consist of four steps documented using a PRISMA flow diagram with reviewer decisions recorded at each stage:

1. The search results will be downloaded onto an excel file and then de-duplicated. Titles with abstracts will be initially screened by at least two reviewers using predefined screening criteria based on whether studies (i) included patients with a diagnosis of a post-viral syndrome and (ii) included information about symptoms, health impacts, and treatments.
2. There will be an assessment of agreement between the reviewers using Cohen's kappa after the screening of 100 titles and abstracts. If there is sufficient agreement between reviewers on article selection (Cohen's kappa >0.9), all further title and abstract screening may continue to be performed by a single reviewer.
3. If there is insufficient agreement between reviewers all remaining titles and abstracts will continue to be selected independently by at least two reviewers and any disagreements resolved through discussion. Where disagreements remain following discussion, these will be reconciled involving an additional reviewer.
4. Full texts of the selected articles will be obtained, and exclusion criteria applied by at least two independent reviewers.
5. As in step 3, selection of included articles will be discussed until a consensus is reached between the reviewers, with an additional reviewer providing support to resolve disagreements. The conclusion of step 5 will provide the final list of studies eligible for inclusion in the review.

The data from all eligible studies will be extracted onto an extraction form (Supplementary File 3) by at least one reviewer, with a quarter of extracted studies checked by a second reviewer. In the event where the full text is not available, we will contact the study's corresponding author for access either via their email address provided for the article or ResearchGate.

Data items

Data will be extracted for all eligible studies including first author, year of publication, study design, study setting (community or hospital), country, participant characteristics (age, sex, ethnicity), name of virus, and outcome measures. A draft of the data extraction tables can be seen in Supplementary file 3.

Outcomes and prioritisation

For peer review only

Data will be sought for the following outcomes: symptoms, health impacts (clinical complications and impacts on quality of life and work capability), and treatments. Each of the four outcomes will be equally prioritised collectively contributing to the primary findings.

Risk of bias in individual studies

To assess the risk of bias in individual studies we will use the appropriate tool for each type of study. AMSTAR-2 will be used for systematic reviews, Cochrane risk of bias tools will be used for randomised (RoB 2) and non-randomised (ROBINS-I) controlled trials, and the Newcastle Ottawa tool will be used for observational studies.[39–42] Two independent reviewers will assess the risk of bias for each individual study and report them using the respective tools.

Data synthesis

The review findings will be presented as two separate reports focusing on: 1) symptoms and health impacts from previous post-viral syndromes, and 2) non-pharmacological treatments for previous post-viral syndromes and Long COVID.

The review will consist of a narrative and quantitative synthesis of included studies if appropriate. The characteristics (study design, population, setting, viruses, etc.) and findings of included studies will first be narratively described and summarised in tables. The outcomes (prevalence of symptoms, health impacts and treatment effects [mean differences or measures of relative risk]) will also be presented on forest plots to assess the heterogeneity of study findings, stratified by virus type. The methodological heterogeneity of studies will be qualitatively evaluated and then assessed using the I^2 statistic.

We will undertake random effects proportion meta-analyses to combine the prevalence of different symptoms and health impacts, where there is sufficient homogeneity between studies. We will also use random effects meta-analysis to combine the treatment effect sizes from RCTs where appropriate. Where there are existing relevant meta-analyses, we will update them using the same approach.

Where outcomes are measured using a continuous scale, these will be pooled through calculation of the weighted mean difference (with standard deviations). Where outcomes are assessed using different continuous measures across the studies, these will be pooled using Hedges' *g* standardised mean difference. Where outcomes are assessed using a dichotomous measure, these will be pooled through calculation of odds ratios.

In line with Cochrane guidance for systematic reviews on dealing with missing data, when appropriate we will not impute missing data but will omit the data from the respective analysis being conducted.[43]

Additional analyses

For peer review only

Where there is sufficient data, we will conduct a subgroup analysis comparing hospitalised to non-hospitalised patients and observing any differences in outcomes. We will also where possible undertake a meta-regression model to assess the impact of health inequalities by age and sex. Additionally, as a sensitivity analysis we will repeat the primary analysis using high quality studies only.

If at least ten studies are included in the meta-analysis with no significant evidence of heterogeneity a funnel plot will be used to explore the existence of publication bias by visual inspection. We will also undertake an objective assessment of publication bias using Egger's regression test. Asymmetry of the funnel plot and/or statistical significance of Egger's regression test ($p<0.05$) will be indicative of publication bias.

Confidence in cumulative evidence

At least two independent reviewers will assess the strength of the body of evidence (according to the following categories: high, moderate, low and very low) for each individual outcome using the five GRADE considerations for downgrading the certainty of evidence (risk of bias, impression, inconsistency, indirectness and publication bias) and the three GRADE considerations for upgrading the certainty of evidence (large magnitude of effect, dose-response gradient and residual confounding decreasing the overall effect).[44]

Patient and public involvement

Patients and the public were involved in the development of the research question and design of the review. This was undertaken with the support of a working patient and public involvement (PPI) group. Patients and the members of the public will not be involved in the study selection or extraction phases but will be involved with the evidence synthesis stage. At this subsequent stage their involvement will be documented according to the GRIPP-2 checklist.[45] The final review, and a lay summary will be disseminated via publication and conference presentations which will be supported with the PPI group.

References

1 John Hopkins University. COVID-19 Map - Johns Hopkins Coronavirus Resource Center. 2020.https://coronavirus.jhu.edu/map.html (accessed 8 Jun2020).

2 Haas EJ, Angulo FJ, McLaughlin JM, Anis E, Singer SR, Khan F *et al.* Impact and effectiveness of mRNA BNT162b2 vaccine against SARS-CoV-2 infections and COVID-19 cases, hospitalisations, and deaths following a nationwide vaccination campaign in Israel: an observational study using national surveillance data. *Lancet* 2021; **397**: 1819–1829.

3 Public Health England. Direct and indirect impact of the vaccination programme on COVID-19 infections and mortality. 2021.

4 Gupta S, Cantor J, Simon KI, Bento AI, Wing C, Whaley CM. Vaccinations Against COVID-19 May Have Averted Up To 140,000 Deaths In The United States. *https://doi.org/101377/hlthaff202100619* 2021; : 10.1377/hlthaff.

For peer review only

5 Daher A, Balfanz P, Cornelissen C, Müller A, Bergs I, Marx N *et al.* Follow up of patients with severe coronavirus disease 2019 (COVID-19): Pulmonary and extrapulmonary disease sequelae. *Respir Med* 2020; **174**. doi:10.1016/j.rmed.2020.106197.

6 Sudre CH, Murray B, Varsavsky T, Graham MS, Penfold RS, Bowyer RC *et al.* Attributes and predictors of Long-COVID: analysis of COVID cases and their symptoms 1 collected by the Covid Symptoms Study App 2 3 doi: medRxiv preprint. *medRxiv* 2020; : 2020.10.19.20214494.

7 Whitaker M, Elliott J, Chadeau-Hyam M, Riley S, Darzi A, Cooke G *et al.* Persistent symptoms following SARS-CoV-2 infection in a random community sample of 508,707 people. 2021.http://spiral.imperial.ac.uk/handle/10044/1/89844 (accessed 10 Sep2021).

8 Office for National Statistics. Short report on Long COVID, 22 July 2021. 2021.https://www.gov.uk/government/publications/ons-short-report-on-long-covid-22-july-2021 (accessed 10 Sep2021).

9 Augustin M, Schommers P, Stecher M, Dewald F, Gieselmann L, Gruell H *et al.* Post-COVID syndrome in non-hospitalised patients with COVID-19: a longitudinal prospective cohort study. *Lancet Reg Heal - Eur* 2021; **6**: 100122.

10 Ayoubkhani D, Khunti K, Nafilyan V, Maddox T, Humberstone B, Diamond I *et al.* Post-covid syndrome in individuals admitted to hospital with covid-19: Retrospective cohort study. *BMJ* 2021; **372**. doi:10.1136/bmj.n693.

11 Blomberg B, Mohn KG-I, Brokstad KA, Zhou F, Linchausen DW, Hansen B-A *et al.* Long COVID in a prospective cohort of home-isolated patients. *Nat Med* 2021; : 1–7.

12 Huang C, Huang L, Wang Y, Li X, Ren L, Gu X *et al.* 6-month consequences of COVID-19 in patients discharged from hospital: a cohort study. *Lancet* 2021; **397**: 220–232.

13 Salamanna F, Veronesi F, Martini L, Landini MP, Fini M. Post-COVID-19 Syndrome: The Persistent Symptoms at the Post-viral Stage of the Disease. A Systematic Review of the Current Data. Front. Med. 2021; **8**: 653516.

14 Aucott JN, Rebman AW. Long-haul COVID: heed the lessons from other infection-triggered illnesses. Lancet. 2021; **397**: 967–968.

15 Gorna R, MacDermott N, Rayner C, O'Hara M, Evans S, Agyen L *et al.* Long COVID guidelines need to reflect lived experience. Lancet. 2021; **397**: 455–457.

16 Norton A, Olliaro P, Sigfrid L, Carson G, Paparella G, Hastie C *et al.* Long COVID: tackling a multifaceted condition requires a multidisciplinary approach. Lancet Infect. Dis. 2021; **21**: 601–602.

17 International Committee on Taxonomy of Viruses. Taxonomy. 2020.https://talk.ictvonline.org/taxonomy/ (accessed 4 Jul2021).

18 Hui DS, Wong KT, Ko FW, Tam LS, Chan DP, Woo J *et al.* The 1-year impact of severe acute respiratory syndrome on pulmonary function, exercise capacity, and quality of life in a cohort of survivors. *Chest* 2005; **128**: 2247–2261.

For peer review only

19 Ong KC, Ng AWK, Lee LSU, Kaw G, Kwek SK, Leow MKS *et al.* 1-Year pulmonary function and health status in survivors of severe acute respiratory syndrome. *Chest* 2005; **128**: 1393–1400.

20 Hsieh M-J, Lee W-C, Cho H-Y, Wu M-F, Hu H-C, Kao K-C *et al.* Recovery of pulmonary functions, exercise capacity, and quality of life after pulmonary rehabilitation in survivors of ARDS due to severe influenza A (H1N1) pneumonitis. *Influenza Other Respi Viruses* 2018; **12**: 643–648.

21 Chen WH, Guo YS, Zhang DH, Zhang HJ. Long-Term Prognosis of Suspected Myocarditis and Cardiomyopathy Associated with Viral Infection of the Myocardial Tissue: A Meta-Analysis of Cohort Studies. *Cardiovasc Ther* 2019; **2019**. doi:10.1155/2019/9342792.

22 Schultz JC, Hilliard AA, Cooper LT, Rihal CS. Diagnosis and Treatment of Viral Myocarditis. *Mayo Clin Proc* 2009; **84**: 1001–1009.

23 Chan KS, Zheng JP, Mok YW, Li YM, Liu YN, Chu CM *et al.* SARS: Prognosis, outcome and sequelae. Respirology. 2003; **8**. doi:10.1046/j.1440-1843.2003.00522.x.

24 Rogers JP, Chesney E, Oliver D, Pollak TA, McGuire P, Fusar-Poli P *et al.* Psychiatric and neuropsychiatric presentations associated with severe coronavirus infections: a systematic review and meta-analysis with comparison to the COVID-19 pandemic. *The Lancet Psychiatry* 2020; **0**. doi:10.1016/S2215-0366(20)30203-0.

25 Lee SH, Shin HS, Park HY, Kim JL, Lee JJ, Lee H *et al.* Depression as a mediator of chronic fatigue and post-traumatic stress symptoms in middle east respiratory syndrome survivors. *Psychiatry Investig* 2019; **16**: 59–64.

26 Hickie I, Davenport T, Wakefield D, Vollmer-Conna U, Cameron B, Vernon SD *et al.* Post-infective and chronic fatigue syndromes precipitated by viral and non-viral pathogens: Prospective cohort study. *Br Med J* 2006; **333**: 575–578.

27 Bansal AS, Bradley AS, Bishop KN, Kiani-Alikhan S, Ford B. Chronic fatigue syndrome, the immune system and viral infection. Brain. Behav. Immun. 2012; **26**: 24–31.

28 Proal A, Marshall T. Myalgic encephalomyelitis/chronic fatigue syndrome in the era of the human microbiome: Persistent pathogens drive chronic symptoms by interfering with host metabolism, gene expression, and immunity. Front. Pediatr. 2018; **6**: 373.

29 Yamato M, Kataoka Y. Fatigue sensation following peripheral viral infection is triggered by neuroinflammation: Who will answer these questions? *Neural Regen Res* 2015; **10**: 203–204.

30 Islam MF, Cotler J, Jason LA. Post-viral fatigue and COVID-19: lessons from past epidemics. *Fatigue Biomed Heal Behav* 2020; **8**: 61–69.

31 Vernon SD, Whistler T, Cameron B, Hickie IB, Reeves WC, Lloyd A. Preliminary evidence of mitochondrial dysfunction associated with post-infective fatigue after acute infection with Epstein Barr Virus. *BMC Infect Dis* 2006; **6**: 15.

32 Holgate ST, Komaroff AL, Mangan D, Wessely S. Chronic fatigue syndrome: Understanding a complex illness. Nat. Rev. Neurosci. 2011; **12**: 539–544.

For peer review only

33 Crook H, Raza S, Nowell J, Young M, Edison P. Long covid—mechanisms, risk factors, and management. *BMJ* 2021; **374**: n1648.

34 National Institute of Health and Care Excellence. Identifying people with ongoing symptomatic COVID-19 or post-COVID-19 syndrome | COVID-19 rapid guideline: managing the long-term effects of COVID-19. 2020.https://www.nice.org.uk/guidance/ng188/chapter/1-Identifying-people-with-ongoing-symptomatic-COVID-19-or-post-COVID-19-syndrome (accessed 10 Sep2021).

35 UKRI. Outcomes of the UK COVID-19 Therapeutics Advisory Panel (UK-CTAP). 2021.https://www.ukri.org/about-us/policies-standards-and-data/data-collection/uk-covid-19-therapeutics-advisory-panel/ (accessed 27 Sep2021).

36 Page MJ, McKenzie JE, Bossuyt PM, Boutron I, Hoffmann TC, Mulrow CD *et al.* The PRISMA 2020 statement: an updated guideline for reporting systematic reviews. *BMJ* 2021; **372**. doi:10.1136/BMJ.N71.

37 Shamseer L, Moher D, Clarke M, Ghersi D, Liberati A, Petticrew M *et al.* Preferred reporting items for systematic review and meta-analysis protocols (prisma-p) 2015: Elaboration and explanation. BMJ. 2015; **349**. doi:10.1136/bmj.g7647.

38 Bennett JE, Dolin R, Blaser MJ. *Principles and practice of infectious diseases*. 2019.

39 Shea BJ, Reeves BC, Wells G, Thuku M, Hamel C, Moran J *et al.* AMSTAR 2: a critical appraisal tool for systematic reviews that include randomised or non-randomised studies of healthcare interventions, or both. *BMJ* 2017; **358**: j4008.

40 Wells G, Shea B, O'Connell D, Peterson J, Welch V, Losos M *et al.* The Newcastle-Ottawa Scale (NOS) for assessing the quality of nonrandomised studies in meta-analyses. Secondary The Newcastle-Ottawa Scale (NOS) for assessing the quality of nonrandomised studies in meta-analyses. *Ottawa Hosp Res Inst* 2015; : 2–4.

41 Sterne JAC, Savović J, Page MJ, Elbers RG, Blencowe NS, Boutron I *et al.* RoB 2: A revised tool for assessing risk of bias in randomised trials. *BMJ* 2019; **366**. doi:10.1136/bmj.l4898.

42 Sterne JA, Hernán MA, Reeves BC, Savović J, Berkman ND, Viswanathan M *et al.* ROBINS-I: A tool for assessing risk of bias in non-randomised studies of interventions. *BMJ* 2016; **355**. doi:10.1136/bmj.i4919.

43 Cochrane. 16.1.2 General principles for dealing with missing data. https://handbook-5-1.cochrane.org/chapter_16/16_1_2_general_principles_for_dealing_with_missing_data.htm (accessed 10 Sep2021).

44 Guyatt GH, Oxman AD, Vist GE, Kunz R, Falck-Ytter Y, Alonso-Coello P *et al.* GRADE: An emerging consensus on rating quality of evidence and strength of recommendations. *BMJ* 2008. doi:10.1136/bmj.39489.470347.ad.

45 Staniszewska S, Brett J, Simera I, Seers K, Mockford C, Goodlad S *et al.* GRIPP2 reporting checklists: Tools to improve reporting of patient and public involvement in research. *BMJ* 2017; **358**: 3453.

For peer review only

Supplementary 1: PRISMA-P checklist

PRISMA-P (Preferred Reporting Items for Systematic review and Meta-Analysis Protocols) checklist: recommended items to address in a systematic review protocol*

Section and topic	Item No	Checklist item	Location in manuscript (page number)
**ADMINISTRATIVE INFORMATION**			
Title:			1
Identification	1a	Identify the report as a protocol of a systematic review	
Update	1b	If the protocol is for an update of a previous systematic review, identify as such	N/A
Registration	2	If registered, provide the name of the registry (such as PROSPERO) and registration number	2
Authors:			1
Contact	3a	Provide name, institutional affiliation, e-mail address of all protocol authors; provide physical mailing address of corresponding author	
Contributions	3b	Describe contributions of protocol authors and identify the guarantor of the review	1
Amendments	4	If the protocol represents an amendment of a previously completed or published protocol, identify as such and list changes; otherwise, state plan for documenting important protocol amendments	N/A
Support:			

For peer review only

Sources	5a	Indicate sources of financial or other support for the review	1
Sponsor	5b	Provide name for the review funder and/or sponsor	1
Role of sponsor or funder	5c	Describe roles of funder(s), sponsor(s), and/or institution(s), if any, in developing the protocol	1
**INTRODUCTION**			
Rationale	6	Describe the rationale for the review in the context of what is already known	4
Objectives	7	Provide an explicit statement of the question(s) the review will address with reference to participants, interventions, comparators, and outcomes (PICO)	5
**METHODS**			
Eligibility criteria	8	Specify the study characteristics (such as PICO, study design, setting, time frame) and report characteristics (such as years considered, language, publication status) to be used as criteria for eligibility for the review	5
Information sources	9	Describe all intended information sources (such as electronic databases, contact with study authors, trial registers or other grey literature sources) with planned dates of coverage	6
Search strategy	10	Present draft of search strategy to be used for at least one electronic database, including planned limits, such that it could be repeated	6 and supplementary table 2
Study records:			
Data management	11a	Describe the mechanism(s) that will be used to manage records and data throughout the review	7

For peer review only

Selection process	11b	State the process that will be used for selecting studies (such as two independent reviewers) through each phase of the review (that is, screening, eligibility and inclusion in meta-analysis)	7
Data collection process	11c	Describe planned method of extracting data from reports (such as piloting forms, done independently, in duplicate), any processes for obtaining and confirming data from investigators	7 and supplementary table 3
Data items	12	List and define all variables for which data will be sought (such as PICO items, funding sources), any pre-planned data assumptions and simplifications	7
Outcomes and prioritization	13	List and define all outcomes for which data will be sought, including prioritization of main and additional outcomes, with rationale	7
Risk of bias in individual studies	14	Describe anticipated methods for assessing risk of bias of individual studies, including whether this will be done at the outcome or study level, or both; state how this information will be used in data synthesis	8
Data synthesis	15a	Describe criteria under which study data will be quantitatively synthesised	8
	15b	If data are appropriate for quantitative synthesis, describe planned summary measures, methods of handling data and methods of combining data from studies, including any planned exploration of consistency (such as I^2, Kendall's τ)	8
	15c	Describe any proposed additional analyses (such as sensitivity or subgroup analyses, meta-regression)	8
	15d	If quantitative synthesis is not appropriate, describe the type of summary planned	8

For peer review only

For peer review only - http://bmjopen.bmj.com/site/about/guidelines.xhtml

Meta-bias(es)	16	Specify any planned assessment of meta-bias(es) (such as publication bias across studies, selective reporting within studies)	8
Confidence in cumulative evidence	17	Describe how the strength of the body of evidence will be assessed (such as GRADE)	9

*** It is strongly recommended that this checklist be read in conjunction with the PRISMA-P Explanation and Elaboration (cite when available) for important clarification on the items. Amendments to a review protocol should be tracked and dated. The copyright for PRISMA-P (including checklist) is held by the PRISMA-P Group and is distributed under a Creative Commons Attribution Licence 4.0.**

For peer review only

Supplementary 2: Draft search terms

Search terms

Virus terms

- Middle eastern respiratory syndrome
  - MERS
- Severe acute respiratory syndrome
  - SARS
- Coronavirus
- Ebola
- Marburg
- Epstein Barr virus
- Cytomegalovirus
- Varicella Zoster Virus
- Other Human Herpes Viruses
- Herpes Simplex Virus
- Influenza
- Influenza A
- Influenza B
- Influenza C
- Influenza D
- Coxsackie
- Hepatitis A
- Enterovirus
- Rhinovirus
- Parvovirus
- Ross River Virus
- Chikungunya
- Semliki Forest Complex Viruses
- West Nile River virus
- Dengue
- Yellow Fever
- Zika
- Rotavirus
- Mumps
- Measles
- Parainfluenza
- Respiratory Syncytial Virus
- Human Metapneumovirus
- Adenoviruses
- Norovirus

For peer review only

- Sapovirus
- Astrovirus*
- COVID

Long-term phrases

- Long-term
- Longterm
- Persistent
- Recurrent
- Chronic
- Post-viral
- Post-infectious
- Post infectious

Symptom terms

- Symptom*
- Fatigue
- Shortness of breath
- Dyspnoea
- Chest pain
- Chest tightness
- Muscle pain
- Myalgia
- Joint pain
- Headache
- Cough
- Chest pain
- Altered smell
- Altered taste
- Diarrhoea
- Brain fog
- Memory loss
- Amnesia
- Palpitations
- Anxiety
- Insomnia
- Sleep disorder*
- Hair loss
- Hot flushes

Health impact terms

- Quality of life
- QoL
- EQ5D

For peer review only

- SF36
- Work capability
- Work ability
- Employment
- Complication
- Clinical complication
- Cardiovascular
- Myocarditis
- Pericarditis
- Cardiac arrythmia
- Arrythmia
- Myocardial inflammation
- Postural orthostatic tachycardia syndrome (POTS)
- Thromboembolic
- Systemic
- Deep vein thrombosis
- DVT
- Renal
- Chronic Kidney Disease
- Renal function
- Microalbuminuria
- Gastrointestinal
- Gastroparesis
- Constipation
- Gastrohepatic
- Diarrhoea
- Gut Dysbiosis
- Neurological
- Post-traumatic stress disorder
- PTSD
- Encephalitis
- Seizures
- Cognitive impairment
- Brain fog
- Tinnitus
- Visual disturbance
- Autonomic dysfunction
- Olfactory dysfunction
- Gustatory Dysfunction
- Respiratory
- Pulmonary embol*
- PE

For peer review only

- Lung function abnormal*
- Lung fibrosis
- DLCO
- Diffusing capacity of the lung for carbon monoxide
- Lung diffusion impairment
- Interstitial thickening
- Pulmonary fibrosis
- Interstitial Lung disease
- Cerebrovascular
- Autoimmune
- Musculoskeletal
- Myositis
- Dermatologic
- Rash
- Inflammatory Skin Conditions
- Cutaneous Vasculitis
- Endocrine
- Endocrinopathy
- Thyroiditis
- Pancreatitis
- Diabetes
- Hypoandrogenism
- Addison’s Disease

Treatment terms

- Treatment*
- Therap*
- Intervention

For peer review only

Supplementary 3: Draft data extraction tables

Outcome of interest: Symptoms and health impacts (clinical complications and impacts on quality of life and work capability)

Author (year)	Study design	Setting	Participant characteristics				Name of virus	Outcomes	Main findings
			N	Mean age, n (SD)	Sex	Ethnicity (%)			

Outcome of interest: treatments

For peer review only - http://bmjopen.bmj.com/site/about/guidelines.xhtml

Author (year)	Setting	Participant characteristics				Name of virus	Outcomes	Intervention	Comparator	Main findings
		N	Mean age, n (SD)	Sex	Ethnicity (%)					

For peer review only

For peer review only - http://bmjopen.bmj.com/site/about/guidelines.xhtml

BMJ Open

Non-pharmacological therapies for post-viral syndromes, including Long COVID: A systematic review and meta-analysis protocol

Journal:	*BMJ Open*
Manuscript ID	bmjopen-2021-057885.R1
Article Type:	Protocol
Date Submitted by the Author:	28-Dec-2021

Complete List of Authors:	Chandan, Joht; University of Birmingham College of Medical and Dental Sciences<br>Brown, Kirsty; University of Birmingham College of Medical and Dental Sciences<br>Simms-Williams, Nikita; University of Birmingham,<br>Camaradou, Jenny; COVID-END<br>Bashir, Nasir; University of Birmingham<br>Heining, Dominic; University Hospitals Birmingham NHS Foundation Trust<br>Aiyegbusi, Olalekan Lee; University of Birmingham, Centre for Patient-Reported Outcomes Research; University of Birmingham, NIHR Birmingham Biomedical Research Centre, University of Birmingham and University Hospitals Birmingham NHS Foundation Trust<br>Turner, Grace; University of Birmingham, Centre for Patient Reported Outcomes Research<br>Cruz Rivera, Samantha; University of Birmingham,<br>Hotham, Richard; University of Birmingham College of Medical and Dental Sciences<br>Nirantharakumar, Krishnarajah; University of Birmingham, Institute of Applied Health Research<br>Sivan, Manoj; University of Leeds,<br>Khunti, Kamlesh; University of Leicester, Department of Health Sciences<br>Raindi, Devan; University of Birmingham<br>Marwaha, Steven; University of Birmingham College of Life and Environmental Sciences<br>Hughes, Sarah; University of Birmingham College of Medical and Dental Sciences<br>McMullan, Christel; University of Birmingham, Institute of Applied Health Research<br>Calvert , Melanie ; University of Birmingham, Centre for Patient Reported Outcomes Research and Institute of Applied Health Research<br>Haroon, Shamil; University of Birmingham,

**Primary Subject Heading**:	Public health
Secondary Subject Heading:	Infectious diseases
Keywords:	COVID-19, REHABILITATION MEDICINE, INFECTIOUS DISEASES

Non-pharmacological therapies for post-viral syndromes, including Long COVID: A systematic review and meta-analysis protocol

Running title: Post-viral syndromes and Long COVID

Authors: Joht Singh Chandan[1]*, Kirsty Brown[1]*, Nikita Simms-Williams[1], Jenny Camaradou[2], Nasir Bashir[3], Dominic Heining[4], Olalekan Lee Aiyegbusi[1,5-8], Grace Turner[1,5], Samantha Cruz Rivera[1,5,7], Richard Hotham[1], Krishnarajah Nirantharakumar[1,12], Manoj Sivam[9], Kamlesh Khunti[10], Devan Raindi[3], Steven Marwaha[11], Sarah E. Hughes[1,5,6], Christel McMullan[1,5], Melanie Calvert[1,5-8,13]†, Shamil Haroon[1]†

*Joint first author
†Joint senior author

Corresponding author:
Dr Joht Singh Chandan
Institute of Applied Health Research, University of Birmingham, Birmingham, UK
Joht.chandan@nhs.net

Affiliations:
[1]Institute of Applied Health Research, University of Birmingham, Birmingham, UK
[2]COVID END Evidence Network
[3]School of Dentistry, University of Birmingham, Birmingham, UK
[4] University Hospitals Birmingham NHS Foundation Trust, Birmingham, UK
[5] Centre for Patient Reported Outcomes Research, Institute of Applied Health Research, University of Birmingham, Birmingham, UK
[6]National Institute for Health Research (NIHR) Applied Research Centre West Midlands, Birmingham, UK
[7]Birmingham Health Partners Centre for Regulatory Science and Innovation, University of Birmingham, Birmingham, UK
[8]NIHR Birmingham Biomedical Research Centre, NIHR Surgical Reconstruction and Microbiology Research Centre, University of Birmingham, Birmingham, UK
[9]School of Medicine, University of Leeds, Leeds, UK
[10]Diabetes Research Centre, University of Leicester, Leicester, UK
[11]Institute for Mental Health, University of Birmingham, Birmingham, UK
[12]Midlands Health Data Research UK, Birmingham, UK

For peer review only
For peer review only - http://bmjopen.bmj.com/site/about/guidelines.xhtml

[13]Health Data Research UK, London, UK

Word count: 2335

Conflict of interest: There are no conflicts of interest among the authors relevant to this study.

Author statement: The research questions were conceived by JSC, KN, MC and SH. The first draft of the protocol was done by KB, NSW, JSC and SH. Further reviews and revisions to the protocol were made by all remaining co-authors (CM, JC, NB, DM, OL, GT, KN, MS, KK, DR, SCR, RH, SM, SEH, KM, JL, DW). The final copy of the manuscript was approved by all authors.

Funding and acknowledgments: This work is independent research jointly funded by the National Institute for Health Research (NIHR) and UK Research and Innovation (UKRI) (Therapies for Long COVID in non-hospitalised individuals: From symptoms, patient reported outcomes and immunology to targeted therapies (The TLC Study), COV-LT-0013). The views expressed in this publication are those of the author(s) and not necessarily those of the National Institute for Health Research or the Department of Health and Social Care.

Data sharing: This protocol does not have any data.

Ethics approval: This study does not require ethical approval as it is a secondary analysis of published data.

Transparency statement: The lead author (the manuscript's guarantor) affirms that this manuscript is an honest, accurate, and transparent account of the study being reported; that no important aspects of the study have been omitted; and that any discrepancies from the study as planned (and, if relevant, registered) have been explained.

For peer review only

Abstract

Introduction*:* Post-viral syndromes (PVS) describe the sustained presence of symptoms following an acute viral infection, for months or even years. Exposure to the SARS-CoV-2 virus and subsequent development of COVID-19 has shown to have similar effects with individuals continuing to exhibit symptoms for greater than twelve weeks. The sustained presence of symptoms is variably referred to as 'post COVID-19 syndrome', 'post-COVID condition' or more commonly 'Long COVID'. Knowledge of the long-term health impacts, and treatments for Long COVID are evolving.

Aims: This review aims to summarise the effectiveness of non-pharmacological treatments for post-viral syndromes, including Long COVID. A secondary aim is to summarise the symptoms and health impacts associated with post-viral syndromes in individuals recruited to treatment studies.

Methods and analysis: Primary electronic searches will be performed in bibliographic databases including: Embase, MEDLINE, PyscINFO, CINAHL and MedRxiv from 1st January 2001 to 29th October 2021. At least two independent reviewers will screen each study for inclusion and data will be extracted from all eligible studies onto a data extraction form. The quality of all included studies will be assessed using AMSTAR-2, Cochrane risk of bias tools and the Newcastle-Ottawa grading system. Non-pharmacological treatments for post-viral syndromes and Long COVID, will be narratively summarised and effect estimates will be pooled using random effects meta-analysis where there is sufficient methodological homogeneity. The symptoms and health impacts reported in the included studies on non-pharmacological interventions will be extracted and narratively reported.

Ethics and dissemination: This systematic review does not require ethical approval. The findings from this study will be submitted for peer-reviewed publication, shared at conference presentations, and disseminated to both clinical and patient groups.

Keywords: Post-viral syndromes, COVID-19, Long COVID, rehabilitation, systematic review

For peer review only - http://bmjopen.bmj.com/site/about/guidelines.xhtml

Strengths and Limitations of this study

- This review will provide the first comprehensive and systematic summary of non-pharmacological treatments for post-viral syndromes.
- The review proposal has been strengthened through its co-development with individuals with lived experience of Long COVID.
- A limitation of the review relates to the heterogeneity in the definition of post-viral syndromes as well as Long COVID, adding complexity to the synthesis of available evidence across the included syndromes.
- There is likely to be significant methodological heterogeneity in clinical trials of non-pharmacological interventions for post-viral syndromes, which may limit our ability to pool treatment effects.

For peer review only

Background

Globally there have been over 275 million cases of COVID-19 with over 5 million associated deaths.[1] The pandemic has triggered a concerted global effort to develop and deliver safe and efficacious vaccinations at record speed, which have now significantly reduced morbidity, mortality, and disease transmission associated with Severe Acute Respiratory Syndrome Coronavirus-2 (SARS CoV-2). [2–4]

Despite such positivity, the long-term sequalae of even mild infections of COVID-19 appear to carry a substantive burden on some patients, their families and health services. Early evidence suggested that the average recovery time following an acute COVID-19 infection is 2-3 weeks, depending on symptom severity.[5,6] However, as the pandemic has progressed it became apparent that not all patients immediately recovered from the initial infection and between 2.3-37% develop symptoms lasting greater than 12 weeks, some of which may be relapsing and remitting.[7,8] However, prevalence estimates in these longer-term symptoms vary because of differences in disease definition (including differences in symptom severity and included symptoms), population surveyed and study design.[8–13] The sustained presence of such symptoms is variably referred to as 'post COVID-19 syndrome' in the UK, 'post-COVID condition' by the World Health Organization or more commonly known as 'Long COVID' (the term used throughout this review) among patient groups.

The prevalence of persistent signs and symptoms, the remitting, relapsing and interchangeable nature of these symptoms, the pathophysiology and clinical course of Long COVID, and the lived experiences of patients, is yet to be fully described. However, many clinical sequalae including, but not limited to, fatigue, respiratory, neurological, psychological and gastroenterological complications that present in patients with Long COVID also feature in a similar pattern following other acute viral infections.[13,14]

An improved understanding of the sequalae and particularly the management of existing post-viral syndromes secondary to acute infections may provide further insight and guidance as to the optimal management of patients with Long COVID. Despite the novelty of the condition, it is clear that optimal management involves a multidisciplinary approach and rehabilitation, with patient involvement being a central component.[15,16]

Post-viral syndromes may occur secondary to exposure to a number of acute viral infections, including but not limited to Coronaviridae, Filoviridae, Herpesviridae, Orthomyxoviridae, Picornaviridae, Parvoviridae, Togaviridae, and Flaviviridae, as defined by the International Committee on Taxonomy of Viruses.[17–25] Like Long COVID, the signs and symptoms comprising these post-viral syndromes may include fatigue, musculoskeletal pain (e.g., joint pain), and mood (e.g. feeling depressed) and neurocognitive disturbances (e.g., difficulties with concentration and sleep).[14,26] A particularly researched area has been the development of post-infectious fatigue following Epstein-Barr virus exposure.[27] As we have demonstrated in our recently published review on the symptoms of Long COVID, fatigue is one of the most commonly reported symptoms.[28] Notably, over time interventions have been developed to

For peer review only - http://bmjopen.bmj.com/site/about/guidelines.xhtml

support patients with Epstein-Barr post-infectious fatigue, such as the combination of cognitive-behavioural therapy with music therapy which has shown some promise.[29] This example highlights the similarities that may exist between post-viral syndromes and the need to summarise evidence on their treatments, which may be relevant to those experiencing Long COVID.

Although the pathophysiology of post-viral syndromes is poorly understood, it has been hypothesised that their development is mediated by altered cytokine responses, neuroinflammation, mitochondrial dysfunction and microbiome dysbiosis. [30–34] A culmination of the pathophysiological changes and phenotypes following acute viral infections, including COVID-19, have been likened to other poorly understood conditions such as myalgic encephalomyelitis (ME)/chronic fatigue syndrome (CFS).[35] Despite the limited evidence and clinical consensus, guidelines for the management of Long COVID have been developed and trials are underway exploring the impact of pharmaceutical, dietary and lifestyle interventions.[36,37]

Considering the significant individual and societal burden arising from the COVID-19 pandemic there is an urgent need to identify whether the existing knowledge base on similar post-viral syndromes can be applied to support the clinical management of patients with Long COVID. A comprehensive synthesis of the evidence on post-viral syndromes, including on their therapies, is needed to better support those with Long COVID as well as those with previous post-viral syndromes.

Objectives

This systematic review will summarise the evidence on non-pharmacological treatments for post-viral syndromes, including Long COVID. The findings will be used to make recommendations on non-pharmacological treatments for Long COVID as well as previous post-viral syndromes. [38]

This systematic review will be conducted and reported in accordance with the PRISMA guidelines, and this protocol has been written in accordance with the PRISMA-P checklist (Supplementary File 1).[39,40] The review will adhere to this protocol which has also been registered with PROSPERO (CRD42021282074).

Methods

Eligibility criteria

We will include primary research studies. For non-pharmacological treatments effects, we will consider randomised and non-randomised controlled trial evidence evaluating interventions for patients with post-viral syndromes and will additionally include observational studies (cohort, case cross-over and case-control study designs) for studies focusing on Long COVID. There will be no restrictions on setting (i.e., community or hospital based). Studies published in the previous 20 years will be included (study period defined as from 1st January 2001 to 29th October 2021), as this would encompass research on the first SARS and MERS outbreak which are the two pandemic viruses most related to SARS-CoV-2.

For peer review only - http://bmjopen.bmj.com/site/about/guidelines.xhtml

No language restrictions will be applied. Peer-reviewed published studies, preprint articles and other grey literature will also be included in this review.

We will include studies with a population consisting of adults and/or children with post-viral syndromes persisting for more than 12 weeks. Studies investigating post-viral syndromes following acute infections from the following families (grouped by the International Committee on Taxonomy of Viruses)[17] will be included in this review.

Viruses known to cause symptomatic acute viral infections will be included. This is based on an assessment of clinically relevant viruses extracted from "Mandell, Douglas, and Bennett's Principles and Practice of Infectious Disease, 9th Edition.[41]" Included in brackets are the relevant clinical infections used to inform search terms:

Coronaviridae (Middle East Respiratory Syndrome-Coronavirus, Severe Acute Respiratory Syndrome-Coronavirus, and other coronaviruses), Filoviridae (Ebola, Marburg), Herpesviridae (Epstein-Barr Virus, Cytomegalovirus, Varicella Zoster Virus, Other Human Herpes Viruses, Herpes Simplex Virus), Orthomyxoviridae (Influenza, A, B, C and D), Picornaviridae (Coxsackie, Hepatitis A, Enterovirus including Rhinovirus), Parvoviridae (Parvovirus), Togaviridae (Ross River virus, Chikungunya, other Semliki Forest Complex Viruses), Flaviviridae (West Nile River Virus, Dengue, Yellow Fever, Zika), Reoviridiae (Rotavirus), Paramyxoviridae (Mumps, Measles, Parainfluenza, Respiratory Syncytial Virus, Human Metapneumovirus) Adenoviridiae (Adenoviruses), Caliciviridae (Norovirus, Sapovirus), Astroviridae (Astroviruses). Additionally, when examining treatment options for Long COVID we will also include studies relating to SARS-CoV-2.

Extremely rare viral infections will not be included, as there is unlikely to be published research relevant to this review. Predominantly asymptomatic viruses, viruses identified in the last 3 years, and viruses predominantly existing as zoonoses will all be excluded in addition to studies on chronic viral infections including Hepatitis B, Hepatitis C and HIV post-viral syndromes. Studies which include demonstrable reactivation of latent infection (e.g EBV, CMV with detectable viral load) will be excluded. We will exclude *in vitro* and animal studies. Studies that principally focus on ME and CFS without reference to pre-specified viruses will also be excluded.

To the authors knowledge there is no internationally agreed definition of post-viral syndromes and as such there is likely to be heterogeneity in the temporal description of syndrome onset following the initial viral exposure. However, similarly to the widely adopted definition of Long COVID, where temporal criteria are included, we will aim to include those with symptoms lasting beyond 12 weeks but will include publications which provide no firm timeframe but indicate an aspect of chronicity or symptoms persisting for a prolonged period. Additionally, like Long COVID, patients with post-viral syndromes can experience a wide range of symptoms ranging from respiratory complications to fatigue or musculoskeletal pain.

The outcomes of the study will be changes in symptoms, exercise capacity, quality of life (including changes in mental health and wellbeing) and work capability . Although, these are

For peer review only - http://bmjopen.bmj.com/site/about/guidelines.xhtml

numerous and heterogenous in nature, some examples of non-pharmacological treatments which may be used to support patients include pacing, cognitive behavioural therapy and pulmonary rehabilitation. Additionally, where described in the included studies, we will also extract information on the symptoms and health impacts reported by participants in the included studies.

In summary, the population will consist of people with a post-viral syndrome (including Long COVID). We will include studies that have assessed the effectiveness of non-pharmacological interventions designed to improve symptoms of post-viral syndromes against standard care, an alternative non-pharmacological therapy, or a placebo. The outcomes will be changes in symptoms, exercise capacity, quality of life (including changes in mental health and wellbeing) and work capability. We will only include randomised controlled trials for studies on post-viral syndromes that predated the COVID-19 pandemic, but we will also include observational studies examining the emerging effectiveness of non-pharmacological interventions for Long COVID.

Information sources

Primary electronic searches will be performed in Embase, MEDLINE, PyscINFO, CINAHL, COVID-NMA and preprint servers (MedRxiv up to the first 500 references) from 1st January 2001 to 29th October 2021. The reference lists of all eligible articles will also be searched to identify any additional eligible studies which were not identified during the original search. Secondary searches will be conducted for grey literature on databases such as Google Scholar (limited to the first 500 references). Abstracts, conference, and symposia proceedings from relevant organisations relating to virology will be identified and examined.

Search methods for identification of studies

The search strategy broadly includes terms such as: “MERS-COV”, “SARS-COV”, “Coronavirus”, “Ebola”, “Epstein-Barr Virus”, “EBV”, “Cytomegalovirus”, “CMV”, “Herpes”, “Influenza”, “Swine flu”, “Coxsackie”, “Parvovirus”, “Ross River virus”, “Chikungunya”, “West Nile River virus”, “long COVID”, “post-acute-COVID”, “Dengue”, “treatment”, “management”, “Post-viral” , “quality of life” , “outcomes”. A more extensive list of search terms is attached in Supplementary File 2.

Study records

Data management, selection, and data collection processes

The study selection procedure will consist of four steps documented using a PRISMA flow diagram with reviewer decisions recorded at each stage:

1. The search results will be downloaded onto an excel file which will be imported onto the online review software COVIDENCE where the results will be de-duplicated.[42] Titles with abstracts will be initially screened by at least two reviewers using predefined screening criteria based on whether studies (i) included patients with a

For peer review only - http://bmjopen.bmj.com/site/about/guidelines.xhtml

diagnosis of a post-viral syndrome and (ii) included information about non-pharmacological treatments.

2. Following title and abstract screening by two reviewers, any disagreements will be resolved through discussion. Where disagreements remain following discussion, these will be reconciled involving an additional reviewer.
3. Full texts of the selected articles will be obtained, and exclusion criteria applied by at least two independent reviewers.
4. As in step 2, selection of included articles will be discussed until a consensus is reached between the reviewers, with an additional reviewer providing support to resolve disagreements. The conclusion of step 3 will provide the final list of studies eligible for inclusion in the review.

The data from all eligible studies will be extracted onto an extraction form by at least one reviewer, with a quarter of extracted studies checked by a second reviewer. In the event where the full text is not available, we will contact the study's corresponding author for access either via their email address provided for the article or ResearchGate.

Data items

Data will be extracted for all eligible studies including first author, year of publication, study design, study setting (community or hospital), country, participant characteristics (age, sex, ethnicity), name of virus, and outcome measures. A draft of the data extraction tables can be seen in Supplementary file 3.

Outcomes and prioritisation

Data will be sought for the type of non-pharmacological treatment/intervention. The outcomes will be changes in symptoms, exercise capacity, quality of life (including changes in mental health and wellbeing) and work capability. Data on the symptoms, health impacts and complications experienced by patients at study entry included in the studies will also be extracted where available.

Risk of bias in individual studies

To assess the risk of bias in individual studies we will use the appropriate tool for each type of study. AMSTAR-2 will be used for systematic reviews, Cochrane risk of bias tools will be used for randomised (RoB 2) and non-randomised (ROBINS-I) controlled trials, and the Newcastle Ottawa tool will be used for observational studies.[43–46] The risk of bias will be examined by two reviewers and reported using the respective tools.

Data synthesis

The review findings will present the non-pharmacological treatments for previous post-viral syndromes and Long COVID.

For peer review only - http://bmjopen.bmj.com/site/about/guidelines.xhtml

The review will consist of a narrative and quantitative synthesis of included studies if appropriate. The characteristics (study design, population, setting, viruses, etc.) and findings of included studies will first be narratively described and summarised in tables. The outcomes (treatment effects [mean differences or measures of relative risk]) will be presented on forest plots to assess the heterogeneity of study findings. The methodological heterogeneity of studies will be qualitatively evaluated and then assessed using the I^2 statistic. We will assess clinical heterogeneity by examining the types of participants, interventions, and outcomes in each study. We anticipate considerable heterogeneity, particularly in exposure definition (post-viral syndromes or Long COVID), due to differing temporal definitions, viral types and nature of symptoms, and thus where meta-analysis is considered appropriate will undertake a random effects meta-analysis rather than a fixed effect model.

Where outcomes are measured using a continuous scale, these will be pooled through calculation of the weighted mean difference (with standard deviations). Where outcomes are assessed using different continuous measures across the studies, these will be pooled using Hedges' *g* standardised mean difference. Where outcomes are assessed using a dichotomous measure, these will be pooled through calculation of odds ratios.

In line with Cochrane guidance for systematic reviews on dealing with missing data, when appropriate we will not impute missing data but will omit the data from the respective analysis being conducted.[47]

Additional analyses

Where there is sufficient data, we will conduct a subgroup analysis comparing hospitalised to non-hospitalised patients and observing any differences in outcomes. We will also where possible undertake a meta-regression model to assess the impact of age and sex on symptoms and treatment effects. Additionally, as a sensitivity analysis we will repeat the primary analysis using high quality studies only.

If at least ten studies are included in the meta-analysis with no significant evidence of heterogeneity a funnel plot will be used to explore the existence of publication bias by visual inspection. We will also undertake an objective assessment of publication bias using Egger's regression test. Asymmetry of the funnel plot and/or statistical significance of Egger's regression test ($p<0.05$) will be indicative of publication bias.

Confidence in cumulative evidence

At least two independent reviewers will assess the strength of the body of evidence (according to the following categories: high, moderate, low and very low) for each individual outcome using the five GRADE considerations for downgrading the certainty of evidence (risk of bias, impression, inconsistency, indirectness and publication bias) and the three GRADE considerations for upgrading the certainty of evidence (large magnitude of effect, dose-response gradient and residual confounding decreasing the overall effect).[48]

For peer review only - http://bmjopen.bmj.com/site/about/guidelines.xhtml

For peer review only

Patient and public involvement

Patients and the public were involved in the development of the research question and design of the review. This was undertaken with the support of a working patient and public involvement and engagement (PPIE) group. We held an initial meeting with members of the PPIE group. Members of the research team gave a presentation which highlighted the similarity between post-viral syndromes and Long COVID, the rationale for this review as well as our preliminary ideas. There were four PPIE group members in attendance – two White men and two women (one Black and the other South-East Asian). Following the presentation, there was a discussion of the review objectives. The PPIE group members were asked for their thoughts on the review objectives and its scope. While they confirmed that the objectives were appropriate and comprehensive, they stressed the impact of long COVID on employment and that the loss of income was very important to patients. Therefore, a key goal of this work should be to identify potential non-pharmacological interventions that will facilitate the rehabilitation of patients with long COVID so that they can be fit to return to work. Based on their feedback, we will ensure that this review will report interventions designed to improve the physical function of patients which will facilitate their return to work. Patients and members of the public will not be involved in the study selection or extraction phases but will be involved with the evidence synthesis stage. At this subsequent stage their involvement will be documented according to the GRIPP-2 checklist.[49] The final review, and a lay summary will be disseminated via publication and conference presentations which will be supported with the PPIE group.

Ethics and dissemination

As the primary focus of the review will be to undertake a secondary analysis of published data, no ethical approval is formally required. The findings from the review will be submitted for peer-reviewed publication, shared at conference presentations, and disseminated to both clinical and patient groups.

References

1 John Hopkins University. COVID-19 Map - Johns Hopkins Coronavirus Resource Center. 2020.https://coronavirus.jhu.edu/map.html (accessed 8 Jun2020).

2 Haas EJ, Angulo FJ, McLaughlin JM, Anis E, Singer SR, Khan F *et al.* Impact and effectiveness of mRNA BNT162b2 vaccine against SARS-CoV-2 infections and COVID-19 cases, hospitalisations, and deaths following a nationwide vaccination campaign in Israel: an observational study using national surveillance data. *Lancet* 2021; **397**: 1819–1829.

3 Public Health England. Direct and indirect impact of the vaccination programme on COVID-19 infections and mortality. 2021.

4 Gupta S, Cantor J, Simon KI, Bento AI, Wing C, Whaley CM. Vaccinations Against COVID-19 May Have Averted Up To 140,000 Deaths In The United States. *https://doi.org/101377/hlthaff202100619* 2021; : 10.1377/hlthaff.

For peer review only - http://bmjopen.bmj.com/site/about/guidelines.xhtml

5 Daher A, Balfanz P, Cornelissen C, Müller A, Bergs I, Marx N *et al.* Follow up of patients with severe coronavirus disease 2019 (COVID-19): Pulmonary and extrapulmonary disease sequelae. *Respir Med* 2020; **174**. doi:10.1016/j.rmed.2020.106197.

6 Sudre CH, Murray B, Varsavsky T, Graham MS, Penfold RS, Bowyer RC *et al.* Attributes and predictors of Long-COVID: analysis of COVID cases and their symptoms 1 collected by the Covid Symptoms Study App 2 3 doi: medRxiv preprint. *medRxiv* 2020; : 2020.10.19.20214494.

7 Whitaker M, Elliott J, Chadeau-Hyam M, Riley S, Darzi A, Cooke G *et al.* Persistent symptoms following SARS-CoV-2 infection in a random community sample of 508,707 people. 2021.http://spiral.imperial.ac.uk/handle/10044/1/89844 (accessed 10 Sep2021).

8 Office for National Statistics. Short report on Long COVID, 22 July 2021. 2021.https://www.gov.uk/government/publications/ons-short-report-on-long-covid-22-july-2021 (accessed 10 Sep2021).

9 Augustin M, Schommers P, Stecher M, Dewald F, Gieselmann L, Gruell H *et al.* Post-COVID syndrome in non-hospitalised patients with COVID-19: a longitudinal prospective cohort study. *Lancet Reg Heal - Eur* 2021; **6**: 100122.

10 Ayoubkhani D, Khunti K, Nafilyan V, Maddox T, Humberstone B, Diamond I *et al.* Post-covid syndrome in individuals admitted to hospital with covid-19: Retrospective cohort study. *BMJ* 2021; **372**. doi:10.1136/bmj.n693.

11 Blomberg B, Mohn KG-I, Brokstad KA, Zhou F, Linchausen DW, Hansen B-A *et al.* Long COVID in a prospective cohort of home-isolated patients. *Nat Med* 2021; : 1–7.

12 Huang C, Huang L, Wang Y, Li X, Ren L, Gu X *et al.* 6-month consequences of COVID-19 in patients discharged from hospital: a cohort study. *Lancet* 2021; **397**: 220–232.

13 Salamanna F, Veronesi F, Martini L, Landini MP, Fini M. Post-COVID-19 Syndrome: The Persistent Symptoms at the Post-viral Stage of the Disease. A Systematic Review of the Current Data. Front. Med. 2021; **8**: 653516.

14 Aucott JN, Rebman AW. Long-haul COVID: heed the lessons from other infection-triggered illnesses. Lancet. 2021; **397**: 967–968.

15 Gorna R, MacDermott N, Rayner C, O'Hara M, Evans S, Agyen L *et al.* Long COVID guidelines need to reflect lived experience. Lancet. 2021; **397**: 455–457.

16 Norton A, Olliaro P, Sigfrid L, Carson G, Paparella G, Hastie C *et al.* Long COVID: tackling a multifaceted condition requires a multidisciplinary approach. Lancet Infect. Dis. 2021; **21**: 601–602.

17 International Committee on Taxonomy of Viruses. Taxonomy. 2020.https://talk.ictvonline.org/taxonomy/ (accessed 4 Jul2021).

18 Hui DS, Wong KT, Ko FW, Tam LS, Chan DP, Woo J *et al.* The 1-year impact of severe acute respiratory syndrome on pulmonary function, exercise capacity, and quality of life in a cohort of survivors. *Chest* 2005; **128**: 2247–2261.

For peer review only - http://bmjopen.bmj.com/site/about/guidelines.xhtml

19 Ong KC, Ng AWK, Lee LSU, Kaw G, Kwek SK, Leow MKS *et al.* 1-Year pulmonary function and health status in survivors of severe acute respiratory syndrome. *Chest* 2005; **128**: 1393–1400.

20 Hsieh M-J, Lee W-C, Cho H-Y, Wu M-F, Hu H-C, Kao K-C *et al.* Recovery of pulmonary functions, exercise capacity, and quality of life after pulmonary rehabilitation in survivors of ARDS due to severe influenza A (H1N1) pneumonitis. *Influenza Other Respi Viruses* 2018; **12**: 643–648.

21 Chen WH, Guo YS, Zhang DH, Zhang HJ. Long-Term Prognosis of Suspected Myocarditis and Cardiomyopathy Associated with Viral Infection of the Myocardial Tissue: A Meta-Analysis of Cohort Studies. *Cardiovasc Ther* 2019; **2019**. doi:10.1155/2019/9342792.

22 Schultz JC, Hilliard AA, Cooper LT, Rihal CS. Diagnosis and Treatment of Viral Myocarditis. *Mayo Clin Proc* 2009; **84**: 1001–1009.

23 Chan KS, Zheng JP, Mok YW, Li YM, Liu YN, Chu CM *et al.* SARS: Prognosis, outcome and sequelae. Respirology. 2003; **8**. doi:10.1046/j.1440-1843.2003.00522.x.

24 Rogers JP, Chesney E, Oliver D, Pollak TA, McGuire P, Fusar-Poli P *et al.* Psychiatric and neuropsychiatric presentations associated with severe coronavirus infections: a systematic review and meta-analysis with comparison to the COVID-19 pandemic. *The Lancet Psychiatry* 2020; **0**. doi:10.1016/S2215-0366(20)30203-0.

25 Lee SH, Shin HS, Park HY, Kim JL, Lee JJ, Lee H *et al.* Depression as a mediator of chronic fatigue and post-traumatic stress symptoms in middle east respiratory syndrome survivors. *Psychiatry Investig* 2019; **16**: 59–64.

26 Hickie I, Davenport T, Wakefield D, Vollmer-Conna U, Cameron B, Vernon SD *et al.* Post-infective and chronic fatigue syndromes precipitated by viral and non-viral pathogens: Prospective cohort study. *Br Med J* 2006; **333**: 575–578.

27 White PD. What Causes Prolonged Fatigue after Infectious Mononucleosis—and Does It Tell Us Anything about Chronic Fatigue Syndrome? *J Infect Dis* 2007; **196**: 4–5.

28 Aiyegbusi OL, Hughes SE, Turner G, Rivera SC, McMullan C, Chandan JS *et al.* Symptoms, complications and management of long COVID: a review. *J R Soc Med* 2021; **114**: 428–442.

29 Malik S, Asprusten TT, Pedersen M, Mangersnes J, Trondalen G, Van Roy B *et al.* Cognitive–behavioural therapy combined with music therapy for chronic fatigue following Epstein-Barr virus infection in adolescents: a randomised controlled trial. *BMJ Paediatr Open* 2020; **4**: e000797.

30 Bansal AS, Bradley AS, Bishop KN, Kiani-Alikhan S, Ford B. Chronic fatigue syndrome, the immune system and viral infection. Brain. Behav. Immun. 2012; **26**: 24–31.

31 Proal A, Marshall T. Myalgic encephalomyelitis/chronic fatigue syndrome in the era of the human microbiome: Persistent pathogens drive chronic symptoms by interfering with host metabolism, gene expression, and immunity. Front. Pediatr. 2018; **6**: 373.

32 Yamato M, Kataoka Y. Fatigue sensation following peripheral viral infection is

For peer review only - http://bmjopen.bmj.com/site/about/guidelines.xhtml

triggered by neuroinflammation: Who will answer these questions? *Neural Regen Res* 2015; **10**: 203–204.

33 Islam MF, Cotler J, Jason LA. Post-viral fatigue and COVID-19: lessons from past epidemics. *Fatigue Biomed Heal Behav* 2020; **8**: 61–69.

34 Vernon SD, Whistler T, Cameron B, Hickie IB, Reeves WC, Lloyd A. Preliminary evidence of mitochondrial dysfunction associated with post-infective fatigue after acute infection with Epstein Barr Virus. *BMC Infect Dis* 2006; **6**: 15.

35 Holgate ST, Komaroff AL, Mangan D, Wessely S. Chronic fatigue syndrome: Understanding a complex illness. Nat. Rev. Neurosci. 2011; **12**: 539–544.

36 Crook H, Raza S, Nowell J, Young M, Edison P. Long covid—mechanisms, risk factors, and management. *BMJ* 2021; **374**: n1648.

37 National Institute of Health and Care Excellence. Identifying people with ongoing symptomatic COVID-19 or post-COVID-19 syndrome | COVID-19 rapid guideline: managing the long-term effects of COVID-19. 2020.https://www.nice.org.uk/guidance/ng188/chapter/1-Identifying-people-with-ongoing-symptomatic-COVID-19-or-post-COVID-19-syndrome (accessed 10 Sep2021).

38 UKRI. Outcomes of the UK COVID-19 Therapeutics Advisory Panel (UK-CTAP). 2021.https://www.ukri.org/about-us/policies-standards-and-data/data-collection/uk-covid-19-therapeutics-advisory-panel/ (accessed 27 Sep2021).

39 Page MJ, McKenzie JE, Bossuyt PM, Boutron I, Hoffmann TC, Mulrow CD *et al.* The PRISMA 2020 statement: an updated guideline for reporting systematic reviews. *BMJ* 2021; **372**. doi:10.1136/BMJ.N71.

40 Shamseer L, Moher D, Clarke M, Ghersi D, Liberati A, Petticrew M *et al.* Preferred reporting items for systematic review and meta-analysis protocols (prisma-p) 2015: Elaboration and explanation. BMJ. 2015; **349**. doi:10.1136/bmj.g7647.

41 Bennett JE, Dolin R, Blaser MJ. *Principles and practice of infectious diseases*. 2019.

42 Veritas Health Innovation. Covidence systematic review software. .

43 Shea BJ, Reeves BC, Wells G, Thuku M, Hamel C, Moran J *et al.* AMSTAR 2: a critical appraisal tool for systematic reviews that include randomised or non-randomised studies of healthcare interventions, or both. *BMJ* 2017; **358**: j4008.

44 Wells G, Shea B, O'Connell D, Peterson J, Welch V, Losos M *et al.* The Newcastle-Ottawa Scale (NOS) for assessing the quality of nonrandomised studies in meta-analyses. Secondary The Newcastle-Ottawa Scale (NOS) for assessing the quality of nonrandomised studies in meta-analyses. *Ottawa Hosp Res Inst* 2015; : 2–4.

45 Sterne JAC, Savović J, Page MJ, Elbers RG, Blencowe NS, Boutron I *et al.* RoB 2: A revised tool for assessing risk of bias in randomised trials. *BMJ* 2019; **366**. doi:10.1136/bmj.l4898.

46 Sterne JA, Hernán MA, Reeves BC, Savović J, Berkman ND, Viswanathan M *et al.* ROBINS-I: A tool for assessing risk of bias in non-randomised studies of interventions.

For peer review only - http://bmjopen.bmj.com/site/about/guidelines.xhtml

BMJ 2016; **355**. doi:10.1136/bmj.i4919.

47 Cochrane. 16.1.2 General principles for dealing with missing data. https://handbook-5-1.cochrane.org/chapter_16/16_1_2_general_principles_for_dealing_with_missing_data.htm (accessed 10 Sep2021).

48 Guyatt GH, Oxman AD, Vist GE, Kunz R, Falck-Ytter Y, Alonso-Coello P *et al.* GRADE: An emerging consensus on rating quality of evidence and strength of recommendations. *BMJ* 2008. doi:10.1136/bmj.39489.470347.ad.

49 Staniszewska S, Brett J, Simera I, Seers K, Mockford C, Goodlad S *et al.* GRIPP2 reporting checklists: Tools to improve reporting of patient and public involvement in research. *BMJ* 2017; **358**: 3453.

For peer review only

For peer review only - http://bmjopen.bmj.com/site/about/guidelines.xhtml

Supplementary 1: PRISMA-P checklist

PRISMA-P (Preferred Reporting Items for Systematic review and Meta-Analysis Protocols) checklist: recommended items to address in a systematic review protocol*

Section and topic	Item No	Checklist item	Location in manuscript (page number)
**ADMINISTRATIVE INFORMATION**			
Title:			1
Identification	1a	Identify the report as a protocol of a systematic review	
Update	1b	If the protocol is for an update of a previous systematic review, identify as such	N/A
Registration	2	If registered, provide the name of the registry (such as PROSPERO) and registration number	6
Authors:			1
Contact	3a	Provide name, institutional affiliation, e-mail address of all protocol authors; provide physical mailing address of corresponding author	
Contributions	3b	Describe contributions of protocol authors and identify the guarantor of the review	1
Amendments	4	If the protocol represents an amendment of a previously completed or published protocol, identify as such and list changes; otherwise, state plan for documenting important protocol amendments	N/A
Support:			

For peer review only

Sources	5a	Indicate sources of financial or other support for the review	1
Sponsor	5b	Provide name for the review funder and/or sponsor	1
Role of sponsor or funder	5c	Describe roles of funder(s), sponsor(s), and/or institution(s), if any, in developing the protocol	1
**INTRODUCTION**			
Rationale	6	Describe the rationale for the review in the context of what is already known	4
Objectives	7	Provide an explicit statement of the question(s) the review will address with reference to participants, interventions, comparators, and outcomes (PICO)	6
**METHODS**			
Eligibility criteria	8	Specify the study characteristics (such as PICO, study design, setting, time frame) and report characteristics (such as years considered, language, publication status) to be used as criteria for eligibility for the review	6
Information sources	9	Describe all intended information sources (such as electronic databases, contact with study authors, trial registers or other grey literature sources) with planned dates of coverage	8
Search strategy	10	Present draft of search strategy to be used for at least one electronic database, including planned limits, such that it could be repeated	8 and supplementary table 2
Study records:			
Data management	11a	Describe the mechanism(s) that will be used to manage records and data throughout the review	8

For peer review only

Selection process	11b	State the process that will be used for selecting studies (such as two independent reviewers) through each phase of the review (that is, screening, eligibility and inclusion in meta-analysis)	8
Data collection process	11c	Describe planned method of extracting data from reports (such as piloting forms, done independently, in duplicate), any processes for obtaining and confirming data from investigators	9 and supplementary table 3
Data items	12	List and define all variables for which data will be sought (such as PICO items, funding sources), any pre-planned data assumptions and simplifications	9
Outcomes and prioritization	13	List and define all outcomes for which data will be sought, including prioritization of main and additional outcomes, with rationale	9
Risk of bias in individual studies	14	Describe anticipated methods for assessing risk of bias of individual studies, including whether this will be done at the outcome or study level, or both; state how this information will be used in data synthesis	9
Data synthesis	15a	Describe criteria under which study data will be quantitatively synthesised	9
	15b	If data are appropriate for quantitative synthesis, describe planned summary measures, methods of handling data and methods of combining data from studies, including any planned exploration of consistency (such as I^2, Kendall's τ)	10
	15c	Describe any proposed additional analyses (such as sensitivity or subgroup analyses, meta-regression)	10
	15d	If quantitative synthesis is not appropriate, describe the type of summary planned	10

For peer review only

Meta-bias(es)	16	Specify any planned assessment of meta-bias(es) (such as publication bias across studies, selective reporting within studies)	10
Confidence in cumulative evidence	17	Describe how the strength of the body of evidence will be assessed (such as GRADE)	10

*** It is strongly recommended that this checklist be read in conjunction with the PRISMA-P Explanation and Elaboration (cite when available) for important clarification on the items. Amendments to a review protocol should be tracked and dated. The copyright for PRISMA-P (including checklist) is held by the PRISMA-P Group and is distributed under a Creative Commons Attribution Licence 4.0.**

For peer review only

Supplementary 2: Draft search terms

Search terms

Virus terms

- Middle eastern respiratory syndrome
  - MERS
- Severe acute respiratory syndrome
  - SARS
- Coronavirus
- Ebola
- Marburg
- Epstein Barr virus
- Cytomegalovirus
- Varicella Zoster Virus
- Other Human Herpes Viruses
- Herpes Simplex Virus
- Influenza
- Influenza A
- Influenza B
- Influenza C
- Influenza D
- Coxsackie
- Hepatitis A
- Enterovirus
- Rhinovirus
- Parvovirus
- Ross River Virus
- Chikungunya
- Semliki Forest Complex Viruses
- West Nile River virus
- Dengue
- Yellow Fever
- Zika
- Rotavirus
- Mumps
- Measles
- Parainfluenza
- Respiratory Syncytial Virus
- Human Metapneumovirus
- Adenoviruses
- Norovirus

For peer review only

- Sapovirus
- Astrovirus*
- COVID

Long-term phrases

- Long-term
- Longterm
- Persistent
- Recurrent
- Chronic
- Post-viral
- Post-infectious
- Post infectious

Treatment terms

- Treatment*
- Therap*
- Intervention

For peer review only

Supplementary 3: Draft data extraction table

Author (year)	Setting	Participant characteristics				Name of virus	Outcomes	Intervention	Comparator	Main findings
		N	Mean age, n (SD)	Sex	Ethnicity (%)					

For peer review only

BMJ Open

Non-pharmacological therapies for post-viral syndromes, including Long COVID: A systematic review and meta-analysis protocol

Journal:	*BMJ Open*
Manuscript ID	bmjopen-2021-057885.R2
Article Type:	Protocol
Date Submitted by the Author:	02-Mar-2022
Complete List of Authors:	Chandan, Joht; University of Birmingham College of Medical and Dental Sciences<br>Brown, Kirsty; University of Birmingham College of Medical and Dental Sciences<br>Simms-Williams, Nikita; University of Birmingham,<br>Camaradou, Jenny; COVID-END<br>Bashir, Nasir; University of Birmingham<br>Heining, Dominic; University Hospitals Birmingham NHS Foundation Trust<br>Aiyegbusi, Olalekan Lee; University of Birmingham, Centre for Patient-Reported Outcomes Research; University of Birmingham, NIHR Birmingham Biomedical Research Centre, University of Birmingham and University Hospitals Birmingham NHS Foundation Trust<br>Turner, Grace; University of Birmingham, Centre for Patient Reported Outcomes Research<br>Cruz Rivera, Samantha; University of Birmingham,<br>Hotham, Richard; University of Birmingham College of Medical and Dental Sciences<br>Nirantharakumar, Krishnarajah; University of Birmingham, Institute of Applied Health Research<br>Sivan, Manoj; University of Leeds,<br>Khunti, Kamlesh; University of Leicester, Department of Health Sciences<br>Raindi, Devan; University of Birmingham<br>Marwaha, Steven; University of Birmingham College of Life and Environmental Sciences<br>Hughes, Sarah; University of Birmingham College of Medical and Dental Sciences<br>McMullan, Christel; University of Birmingham, Institute of Applied Health Research<br>Calvert , Melanie ; University of Birmingham, Centre for Patient Reported Outcomes Research and Institute of Applied Health Research<br>Haroon, Shamil; University of Birmingham,
**Primary Subject Heading**:	Public health
Secondary Subject Heading:	Infectious diseases
Keywords:	COVID-19, REHABILITATION MEDICINE, INFECTIOUS DISEASES

For peer review only - http://bmjopen.bmj.com/site/about/guidelines.xhtml

Non-pharmacological therapies for post-viral syndromes, including Long COVID: A systematic review and meta-analysis protocol

Running title: Post-viral syndromes and Long COVID

Authors: Joht Singh Chandan[1]*, Kirsty Brown[1]*, Nikita Simms-Williams[1], Jenny Camaradou[2], Nasir Bashir[3], Dominic Heining[4], Olalekan Lee Aiyegbusi[1,5-8], Grace Turner[1,5], Samantha Cruz Rivera[1,5,7], Richard Hotham[1], Krishnarajah Nirantharakumar[1,12], Manoj Sivam[9], Kamlesh Khunti[10], Devan Raindi[3], Steven Marwaha[11], Sarah E. Hughes[1,5,6], Christel McMullan[1,5], Melanie Calvert[1,5-8,13]†, Shamil Haroon[1]†

*Joint first author
†Joint senior author

Corresponding author:
Dr Joht Singh Chandan
Institute of Applied Health Research, University of Birmingham, Birmingham, UK
Joht.chandan@nhs.net

Affiliations:
[1]Institute of Applied Health Research, University of Birmingham, Birmingham, UK
[2]COVID END Evidence Network
[3]School of Dentistry, University of Birmingham, Birmingham, UK
[4] University Hospitals Birmingham NHS Foundation Trust, Birmingham, UK
[5] Centre for Patient Reported Outcomes Research, Institute of Applied Health Research, University of Birmingham, Birmingham, UK
[6]National Institute for Health Research (NIHR) Applied Research Centre West Midlands, Birmingham, UK
[7]Birmingham Health Partners Centre for Regulatory Science and Innovation, University of Birmingham, Birmingham, UK
[8]NIHR Birmingham Biomedical Research Centre, NIHR Surgical Reconstruction and Microbiology Research Centre, University of Birmingham, Birmingham, UK
[9]School of Medicine, University of Leeds, Leeds, UK
[10]Diabetes Research Centre, University of Leicester, Leicester, UK
[11]Institute for Mental Health, University of Birmingham, Birmingham, UK
[12]Midlands Health Data Research UK, Birmingham, UK

For peer review only - http://bmjopen.bmj.com/site/about/guidelines.xhtml

[13]Health Data Research UK, London, UK

Word count: 2335

Conflict of interest: There are no conflicts of interest among the authors relevant to this study.

Author statement: The research questions were conceived by JSC, KN, MC and SH. The first draft of the protocol was done by KB, NSW, JSC and SH. Further reviews and revisions to the protocol were made by all remaining co-authors (CM, JC, NB, DM, OL, GT, KN, MS, KK, DR, SCR, RH, SM, SEH, KM, JL, DW). The final copy of the manuscript was approved by all authors.

Funding and acknowledgments: This work is independent research jointly funded by the National Institute for Health Research (NIHR) and UK Research and Innovation (UKRI) (Therapies for Long COVID in non-hospitalised individuals: From symptoms, patient reported outcomes and immunology to targeted therapies (The TLC Study), COV-LT-0013). The views expressed in this publication are those of the author(s) and not necessarily those of the National Institute for Health Research or the Department of Health and Social Care.

Data sharing: This protocol does not have any data.

Ethics approval: This study does not require ethical approval as it is a secondary analysis of published data.

Transparency statement: The lead author (the manuscript's guarantor) affirms that this manuscript is an honest, accurate, and transparent account of the study being reported; that no important aspects of the study have been omitted; and that any discrepancies from the study as planned (and, if relevant, registered) have been explained.

For peer review only

Abstract

Introduction: Post-viral syndromes (PVS) describe the sustained presence of symptoms following an acute viral infection, for months or even years. Exposure to the SARS-CoV-2 virus and subsequent development of COVID-19 has shown to have similar effects with individuals continuing to exhibit symptoms for greater than twelve weeks. The sustained presence of symptoms is variably referred to as 'post COVID-19 syndrome', 'post-COVID condition' or more commonly 'Long COVID'. Knowledge of the long-term health impacts, and treatments for Long COVID are evolving. To minimise overlap with existing work in the field exploring treatments of Long COVID, we have only chosen to focus on non-pharmacological treatments.

Aims: This review aims to summarise the effectiveness of non-pharmacological treatments for post-viral syndromes, including Long COVID. A secondary aim is to summarise the symptoms and health impacts associated with post-viral syndromes in individuals recruited to treatment studies.

Methods and analysis: Primary electronic searches will be performed in bibliographic databases including: Embase, MEDLINE, PyscINFO, CINAHL and MedRxiv from 1st January 2001 to 29th October 2021. At least two independent reviewers will screen each study for inclusion and data will be extracted from all eligible studies onto a data extraction form. The quality of all included studies will be assessed using Cochrane risk of bias tools and the Newcastle-Ottawa grading system. Non-pharmacological treatments for post-viral syndromes and Long COVID will be narratively summarised and effect estimates will be pooled using random effects meta-analysis where there is sufficient methodological homogeneity. The symptoms and health impacts reported in the included studies on non-pharmacological interventions will be extracted and narratively reported.

Ethics and dissemination: This systematic review does not require ethical approval. The findings from this study will be submitted for peer-reviewed publication, shared at conference presentations, and disseminated to both clinical and patient groups.

Keywords: Post-viral syndromes, COVID-19, Long COVID, rehabilitation, systematic review

For peer review only - http://bmjopen.bmj.com/site/about/guidelines.xhtml

Strengths and Limitations of this study

- This review will provide the first comprehensive and systematic summary of non-pharmacological treatments for post-viral syndromes.
- The review proposal has been strengthened through its co-development with individuals with lived experience of Long COVID.
- A limitation of the review relates to the heterogeneity in the definition of post-viral syndromes as well as Long COVID, adding complexity to the synthesis of available evidence across the included syndromes.
- There is likely to be significant methodological heterogeneity in clinical trials of non-pharmacological interventions for post-viral syndromes, which may limit our ability to pool treatment effects.

For peer review only

Background

Globally there have been over 275 million cases of COVID-19 with over 5 million associated deaths.[1] The pandemic has triggered a concerted global effort to develop and deliver safe and efficacious vaccinations at record speed, which have now significantly reduced morbidity, mortality, and disease transmission associated with Severe Acute Respiratory Syndrome Coronavirus-2 (SARS CoV-2). [2–4]

Despite such positivity, the long-term sequalae of even mild infections of COVID-19 appear to carry a substantive burden on some patients, their families and health services. Early evidence suggested that the average recovery time following an acute COVID-19 infection is 2-3 weeks, depending on symptom severity.[5,6] However, as the pandemic has progressed it became apparent that not all patients immediately recovered from the initial infection and between 2.3-37% develop symptoms lasting greater than 12 weeks, some of which may be relapsing and remitting.[7,8] However, prevalence estimates in these longer-term symptoms vary because of differences in disease definition (including differences in symptom severity and included symptoms), population surveyed and study design.[8–13]

There is considerable heterogeneity in the clinical definition of ongoing symptoms following initial exposure to SARS CoV-2. The National Institute for Health and Care Excellence (NICE) define symptoms persisting beyond 4 weeks as either 'ongoing symptomatic COVID-19' (symptoms lasting between 4-12 weeks) or 'Post covid-19 syndrome' (symptoms lasting beyond 12 weeks).[14] Globally other terms such as 'post-COVID condition' or 'long haulers' are used. However, in the UK the more commonly adopted and preferred term among healthcare professionals and patient groups for ongoing symptoms following initial viral exposure to SARS CoV-2 is 'Long COVID,' the term adopted for this review.

The prevalence of persistent signs and symptoms, the remitting, relapsing and interchangeable nature of these symptoms, the pathophysiology and clinical course of Long COVID, and the lived experiences of patients, is yet to be fully described. However, many clinical sequalae including, but not limited to, fatigue, respiratory, neurological, psychological and gastroenterological complications that present in patients with Long COVID also feature in a similar pattern following other acute viral infections.[13,15]

An improved understanding of the sequalae and particularly the management of existing post-viral syndromes secondary to acute infections may provide further insight and guidance as to the optimal management of patients with Long COVID. Despite the novelty of the condition, it is clear that optimal management involves a multidisciplinary approach and rehabilitation, with patient involvement being a central component.[16,17]

Post-viral syndromes may occur secondary to exposure to a number of acute viral infections, including but not limited to Coronaviridae, Filoviridae, Herpesviridae, Orthomyxoviridae,

For peer review only - http://bmjopen.bmj.com/site/about/guidelines.xhtml

Picornaviridae, Parvoviridae, Togaviridae, and Flaviviridae, as defined by the International Committee on Taxonomy of Viruses.[18–26] Like Long COVID, the signs and symptoms comprising these post-viral syndromes may include fatigue, musculoskeletal pain (e.g., joint pain), and mood (e.g. feeling depressed) and neurocognitive disturbances (e.g., difficulties with concentration and sleep).[15,27] A particularly researched area has been the development of post-infectious fatigue following Epstein-Barr virus exposure.[28] As we have demonstrated in our recently published review on the symptoms of Long COVID, fatigue is one of the most commonly reported symptoms.[29] Notably, over time interventions have been developed to support patients with Epstein-Barr post-infectious fatigue, such as the combination of cognitive-behavioural therapy with music therapy which has shown some promise.[30] This example highlights the similarities that may exist between post-viral syndromes and the need to summarise evidence on their treatments, which may be relevant to those experiencing Long COVID.

Although the pathophysiology of post-viral syndromes is poorly understood, it has been hypothesised that their development is mediated by altered cytokine responses, neuroinflammation, mitochondrial dysfunction and microbiome dysbiosis.[31–35] A culmination of the pathophysiological changes and phenotypes following acute viral infections, including COVID-19, have been likened to other poorly understood conditions such as myalgic encephalomyelitis (ME)/chronic fatigue syndrome (CFS).[36] Despite the limited evidence and clinical consensus, guidelines for the management of Long COVID have been developed and trials are underway exploring the impact of pharmaceutical, dietary and lifestyle interventions.[14,37]

Considering the significant individual and societal burden arising from the COVID-19 pandemic there is an urgent need to identify whether the existing knowledge base on similar post-viral syndromes can be applied to support the clinical management of patients with Long COVID. A comprehensive synthesis of the evidence on post-viral syndromes, including on their therapies, is needed to better support those with Long COVID as well as those with previous post-viral syndromes. To minimise overlap with existing work in the field exploring pharmacological treatments for Long COVID and on the basis of the advice of our expert advisory group, we have chosen to focus on non-pharmacological treatments.

Objectives

This systematic review will summarise the evidence on non-pharmacological treatments for post-viral syndromes, including Long COVID. The findings will be used to make recommendations on non-pharmacological treatments for Long COVID as well as previous post-viral syndromes.[38]

This systematic review will be conducted and reported in accordance with the PRISMA guidelines, and this protocol has been written in accordance with the PRISMA-P checklist (Supplementary File 1).[39,40] The review will adhere to this protocol which has also been registered with PROSPERO (CRD42021282074).

For peer review only - http://bmjopen.bmj.com/site/about/guidelines.xhtml

Methods

Eligibility criteria

We will include primary research studies. For non-pharmacological treatments effects, we will consider randomised and non-randomised controlled trial evidence evaluating interventions for patients with post-viral syndromes and will additionally include observational studies (cohort, case cross-over and case-control study designs) for studies focusing on Long COVID. There will be no restrictions on setting (i.e., community or hospital based). Studies published in the previous 20 years will be included (study period defined as from 1st January 2001 to 29th October 2021), as this would encompass research on the first SARS and MERS outbreak which are the two pandemic viruses most related to SARS-CoV-2. No language restrictions will be applied. Peer-reviewed published studies, preprint articles and other grey literature will also be included in this review.

We will include studies with a population consisting of adults and/or children with post-viral syndromes. Studies investigating post-viral syndromes following acute infections from the following families (grouped by the International Committee on Taxonomy of Viruses)[18] will be included in this review.

Viruses known to cause symptomatic acute viral infections will be included. This is based on an assessment of clinically relevant viruses extracted from "Mandell, Douglas, and Bennett's Principles and Practice of Infectious Disease, 9th Edition."[41] Included in brackets are the relevant clinical infections used to inform search terms:

Coronaviridae (Middle East Respiratory Syndrome-Coronavirus, Severe Acute Respiratory Syndrome-Coronavirus, and other coronaviruses), Filoviridae (Ebola, Marburg), Herpesviridae (Epstein-Barr Virus, Cytomegalovirus, Varicella Zoster Virus, Other Human Herpes Viruses, Herpes Simplex Virus), Orthomyxoviridae (Influenza, A, B, C and D), Picornaviridae (Coxsackie, Hepatitis A, Enterovirus including Rhinovirus), Parvoviridae (Parvovirus), Togaviridae (Ross River virus, Chikungunya, other Semliki Forest Complex Viruses), Flaviviridae (West Nile River Virus, Dengue, Yellow Fever, Zika), Reoviridiae (Rotavirus), Paramyxoviridae (Mumps, Measles, Parainfluenza, Respiratory Syncytial Virus, Human Metapneumovirus) Adenoviridiae (Adenoviruses), Caliciviridae (Norovirus, Sapovirus), Astroviridae (Astroviruses). Additionally, when examining treatment options for Long COVID we will also include studies relating to SARS-CoV-2.

Extremely rare viral infections will not be included, as there is unlikely to be published research relevant to this review. Predominantly asymptomatic viruses, viruses identified in the last 3 years (except for SARS CoV-2), and viruses predominantly existing as zoonoses will all be excluded, in addition to studies on chronic viral infections including Hepatitis B, Hepatitis C and HIV post-viral syndromes. Studies which include demonstrable reactivation of latent infection (e.g EBV, CMV with detectable viral load) will be excluded. We will exclude *in vitro* and animal studies. Studies that principally focus on ME and CFS without reference to pre-specified viruses will also be excluded.

For peer review only - http://bmjopen.bmj.com/site/about/guidelines.xhtml

To the authors' knowledge there is no internationally agreed definition of post-viral syndromes and as such there is likely to be heterogeneity in the temporal description of syndrome onset following the initial viral exposure. Where temporal criteria are included, we will aim to include those with symptoms lasting beyond 12 weeks (in line with the minimum timeframe for Long COVID used by the World Health Organization (WHO)).[42] However we will also include publications which provide no firm timeframe but indicate an aspect of chronicity or symptoms persisting for a prolonged period. Additionally, like Long COVID, patients with post-viral syndromes can experience a wide range of symptoms ranging from respiratory complications to fatigue or musculoskeletal pain.

The outcomes of the study will be changes in symptoms, exercise capacity, quality of life (including changes in mental health and wellbeing) and work capability. Although these are numerous and heterogenous in nature, some examples of non-pharmacological treatments which may be used to support patients include pacing, cognitive behavioural therapy and pulmonary rehabilitation. Additionally, where described in the included studies, we will also extract information on the symptoms and health impacts reported by participants in the included studies.

In summary, the population will consist of either people with a post-viral syndrome who contributed to a randomised controlled trial or those with Long COVID who either contributed to an observational study or a randomised controlled trial. We will include studies that have assessed the effectiveness of non-pharmacological interventions designed to improve symptoms of post-viral syndromes against standard care, an alternative non-pharmacological therapy, or a placebo. The outcomes will be changes in symptoms, exercise capacity, quality of life (including changes in mental health and wellbeing), and work capability.

Information sources

Primary electronic searches will be performed in Embase, MEDLINE, PyscINFO, CINAHL, COVID-NMA and preprint servers (MedRxiv up to the first 500 references) from 1st January 2001 to 29th October 2021. The reference lists of all eligible articles will also be searched to identify any additional eligible studies which were not identified during the original search. Secondary searches will be conducted for grey literature on databases such as Google Scholar (limited to the first 500 references). Abstracts, conference, and symposia proceedings from relevant organisations relating to virology will be identified and examined.

Search methods for identification of studies

The search strategy broadly includes terms such as: "MERS-COV", "SARS-COV", "Coronavirus", "Ebola", "Epstein-Barr Virus", "EBV", "Cytomegalovirus", "CMV", "Herpes", "Influenza", "Swine flu", "Coxsackie", "Parvovirus", "Ross River virus", "Chikungunya", "West Nile River virus", "long COVID", "post-acute-COVID", "Dengue", "treatment", "management", "Post-viral" , "quality of life" , "outcomes". A more extensive list of search terms is attached in Supplementary File 2.

For peer review only - http://bmjopen.bmj.com/site/about/guidelines.xhtml

Studies will not be excluded on language. Where studies are not published in English then they will be translated using Google Translate or a by a member of staff at the Institute of Applied Health Research.

Study records

Data management, selection, and data collection processes

The study selection procedure will consist of four steps documented using a PRISMA flow diagram with reviewer decisions recorded at each stage:

1. The search results will be downloaded onto an excel file which will be imported onto the online review software COVIDENCE where the results will be de-duplicated.[43] Titles with abstracts will be initially screened by at least two reviewers using predefined screening criteria based on whether studies (i) included patients with a diagnosis of a post-viral syndrome and (ii) included information about non-pharmacological treatments.
2. Following title and abstract screening by two reviewers, any disagreements will be resolved through discussion. Where disagreements remain following discussion, these will be reconciled involving an additional reviewer.
3. Full texts of the selected articles will be obtained, and exclusion criteria applied by at least two independent reviewers.
4. As in step 2, selection of included articles will be discussed until a consensus is reached between the reviewers, with an additional reviewer providing support to resolve disagreements. The conclusion of step 3 will provide the final list of studies eligible for inclusion in the review.

The data from all eligible studies will be extracted onto an extraction form by at least one reviewer, with a quarter of extracted studies checked by a second reviewer. In the event where the full text is not available, we will contact the study's corresponding author for access either via their email address provided for the article or ResearchGate.

Data items

Data will be extracted for all eligible studies including first author, year of publication, study design, study setting (community or hospital), country, participant characteristics (age, sex, ethnicity), name of virus, and outcome measures. A draft of the data extraction tables can be seen in Supplementary file 3.

Outcomes and prioritisation

Data will be sought for the type of non-pharmacological treatment/intervention. The outcomes will be changes in symptoms, exercise capacity, quality of life (including changes in mental health and wellbeing), and work capability. Data on the symptoms, health impacts and complications experienced by patients at study entry included in the studies will also be extracted where available.

For peer review only - http://bmjopen.bmj.com/site/about/guidelines.xhtml

Risk of bias in individual studies

To assess the risk of bias in individual studies we will use the appropriate tool for each type of study. Cochrane risk of bias tools will be used for randomised (RoB 2) and non-randomised (ROBINS-I) controlled trials, and the Newcastle Ottawa tool will be used for observational studies.[44–47] The risk of bias will be examined by two reviewers and reported using the respective tools.

Data synthesis

The review findings will present the non-pharmacological treatments for previous post-viral syndromes and Long COVID.

The review will consist of a narrative and quantitative synthesis of included studies if appropriate. The characteristics (study design, population, setting, viruses, etc.) and findings of included studies will first be narratively described and summarised in tables. The outcomes (treatment effects [mean differences or measures of relative risk]) will be presented on forest plots to assess the heterogeneity of study findings. The methodological heterogeneity of studies will be qualitatively evaluated and then assessed using the I^2 statistic. We will assess clinical heterogeneity by examining the types of participants, interventions, and outcomes in each study. We anticipate considerable heterogeneity, particularly in exposure definition (post-viral syndromes or Long COVID), due to differing temporal definitions, viral types and nature of symptoms, and thus where meta-analysis is considered appropriate will undertake a random effects meta-analysis rather than a fixed effect model.

Where outcomes are measured using a continuous scale, these will be pooled through calculation of the weighted mean difference (with standard deviations). Where outcomes are assessed using different continuous measures across the studies, these will be pooled using Hedges' *g* standardised mean difference. Where outcomes are assessed using a dichotomous measure, these will be pooled through calculation of odds ratios.

In line with Cochrane guidance for systematic reviews on dealing with missing data, when appropriate we will not impute missing data but will omit the data from the respective analysis being conducted.[48]

Additional analyses

Where there is sufficient data, we will conduct a subgroup analysis comparing hospitalised to non-hospitalised patients and observing any differences in outcomes. We will also where possible undertake a meta-regression model to assess the impact of age and sex on symptoms and treatment effects. Additionally, as a sensitivity analysis we will repeat the primary analysis using high quality studies only.

If at least ten studies are included in the meta-analysis with no significant evidence of heterogeneity a funnel plot will be used to explore the existence of publication bias by visual

For peer review only - http://bmjopen.bmj.com/site/about/guidelines.xhtml

inspection. We will also undertake an objective assessment of publication bias using Egger's regression test. Asymmetry of the funnel plot and/or statistical significance of Egger's regression test ($p<0.05$) will be indicative of publication bias.

Confidence in cumulative evidence

At least two independent reviewers will assess the strength of the body of evidence (according to the following categories: high, moderate, low and very low) for each individual outcome using the five GRADE considerations for downgrading the certainty of evidence (risk of bias, impression, inconsistency, indirectness and publication bias) and the three GRADE considerations for upgrading the certainty of evidence (large magnitude of effect, dose-response gradient and residual confounding decreasing the overall effect).[49]

Patient and public involvement

Patients and the public were involved in the development of the research question and design of the review. This was undertaken with the support of a working patient and public involvement and engagement (PPIE) group. We held an initial meeting with members of the PPIE group. Members of the research team gave a presentation which highlighted the similarity between post-viral syndromes and Long COVID, the rationale for this review as well as our preliminary ideas. There were four PPIE group members in attendance – two White men and two women (one Black and the other South-East Asian). Following the presentation, there was a discussion of the review objectives. The PPIE group members were asked for their thoughts on the review objectives and its scope. While they confirmed that the objectives were appropriate and comprehensive, they stressed the impact of Long COVID on employment and that the loss of income was very important to patients. Therefore, a key goal of this work should be to identify potential non-pharmacological interventions that will facilitate the rehabilitation of patients with long COVID so that they can be fit to return to work. Based on their feedback, we will ensure that this review will report interventions designed to improve the physical function of patients which will facilitate their return to work. Patients and members of the public will not be involved in the study selection or extraction phases but will be involved with the evidence synthesis stage. At this subsequent stage their involvement will be documented according to the GRIPP-2 checklist.[50] The final review, and a lay summary will be disseminated via publication and conference presentations which will be supported with the PPIE group.

Ethics and dissemination

As the primary focus of the review will be to undertake a secondary analysis of published data, no ethical approval is formally required. The findings from the review will be submitted for peer-reviewed publication, shared at conference presentations, and disseminated to both clinical and patient groups.

References

1 John Hopkins University. COVID-19 Map - Johns Hopkins Coronavirus Resource

For peer review only - http://bmjopen.bmj.com/site/about/guidelines.xhtml

For peer review only

Center. 2020.https://coronavirus.jhu.edu/map.html (accessed 8 Jun2020).

2 Haas EJ, Angulo FJ, McLaughlin JM, Anis E, Singer SR, Khan F *et al.* Impact and effectiveness of mRNA BNT162b2 vaccine against SARS-CoV-2 infections and COVID-19 cases, hospitalisations, and deaths following a nationwide vaccination campaign in Israel: an observational study using national surveillance data. *Lancet* 2021; **397**: 1819–1829.

3 Public Health England. Direct and indirect impact of the vaccination programme on COVID-19 infections and mortality. 2021.

4 Gupta S, Cantor J, Simon KI, Bento AI, Wing C, Whaley CM. Vaccinations Against COVID-19 May Have Averted Up To 140,000 Deaths In The United States. *https://doi.org/101377/hlthaff202100619* 2021; : 10.1377/hlthaff.

5 Daher A, Balfanz P, Cornelissen C, Müller A, Bergs I, Marx N *et al.* Follow up of patients with severe coronavirus disease 2019 (COVID-19): Pulmonary and extrapulmonary disease sequelae. *Respir Med* 2020; **174**. doi:10.1016/j.rmed.2020.106197.

6 Sudre CH, Murray B, Varsavsky T, Graham MS, Penfold RS, Bowyer RC *et al.* Attributes and predictors of Long-COVID: analysis of COVID cases and their symptoms 1 collected by the Covid Symptoms Study App 2 3 doi: medRxiv preprint. *medRxiv* 2020; : 2020.10.19.20214494.

7 Whitaker M, Elliott J, Chadeau-Hyam M, Riley S, Darzi A, Cooke G *et al.* Persistent symptoms following SARS-CoV-2 infection in a random community sample of 508,707 people. 2021.http://spiral.imperial.ac.uk/handle/10044/1/89844 (accessed 10 Sep2021).

8 Office for National Statistics. Short report on Long COVID, 22 July 2021. 2021.https://www.gov.uk/government/publications/ons-short-report-on-long-covid-22-july-2021 (accessed 10 Sep2021).

9 Augustin M, Schommers P, Stecher M, Dewald F, Gieselmann L, Gruell H *et al.* Post-COVID syndrome in non-hospitalised patients with COVID-19: a longitudinal prospective cohort study. *Lancet Reg Heal - Eur* 2021; **6**: 100122.

10 Ayoubkhani D, Khunti K, Nafilyan V, Maddox T, Humberstone B, Diamond I *et al.* Post-covid syndrome in individuals admitted to hospital with covid-19: Retrospective cohort study. *BMJ* 2021; **372**. doi:10.1136/bmj.n693.

11 Blomberg B, Mohn KG-I, Brokstad KA, Zhou F, Linchausen DW, Hansen B-A *et al.* Long COVID in a prospective cohort of home-isolated patients. *Nat Med* 2021; : 1–7.

12 Huang C, Huang L, Wang Y, Li X, Ren L, Gu X *et al.* 6-month consequences of COVID-19 in patients discharged from hospital: a cohort study. *Lancet* 2021; **397**: 220–232.

13 Salamanna F, Veronesi F, Martini L, Landini MP, Fini M. Post-COVID-19 Syndrome: The Persistent Symptoms at the Post-viral Stage of the Disease. A Systematic Review of the Current Data. Front. Med. 2021; **8**: 653516.

14 National Institute of Health and Care Excellence. Identifying people with ongoing

For peer review only - http://bmjopen.bmj.com/site/about/guidelines.xhtml

symptomatic COVID-19 or post-COVID-19 syndrome | COVID-19 rapid guideline: managing the long-term effects of COVID-19. 2020.https://www.nice.org.uk/guidance/ng188/chapter/1-Identifying-people-with-ongoing-symptomatic-COVID-19-or-post-COVID-19-syndrome (accessed 10 Sep2021).

15 Aucott JN, Rebman AW. Long-haul COVID: heed the lessons from other infection-triggered illnesses. Lancet. 2021; **397**: 967–968.

16 Gorna R, MacDermott N, Rayner C, O'Hara M, Evans S, Agyen L *et al.* Long COVID guidelines need to reflect lived experience. Lancet. 2021; **397**: 455–457.

17 Norton A, Olliaro P, Sigfrid L, Carson G, Paparella G, Hastie C *et al.* Long COVID: tackling a multifaceted condition requires a multidisciplinary approach. Lancet Infect. Dis. 2021; **21**: 601–602.

18 International Committee on Taxonomy of Viruses. Taxonomy. 2020.https://talk.ictvonline.org/taxonomy/ (accessed 4 Jul2021).

19 Hui DS, Wong KT, Ko FW, Tam LS, Chan DP, Woo J *et al.* The 1-year impact of severe acute respiratory syndrome on pulmonary function, exercise capacity, and quality of life in a cohort of survivors. *Chest* 2005; **128**: 2247–2261.

20 Ong KC, Ng AWK, Lee LSU, Kaw G, Kwek SK, Leow MKS *et al.* 1-Year pulmonary function and health status in survivors of severe acute respiratory syndrome. *Chest* 2005; **128**: 1393–1400.

21 Hsieh M-J, Lee W-C, Cho H-Y, Wu M-F, Hu H-C, Kao K-C *et al.* Recovery of pulmonary functions, exercise capacity, and quality of life after pulmonary rehabilitation in survivors of ARDS due to severe influenza A (H1N1) pneumonitis. *Influenza Other Respi Viruses* 2018; **12**: 643–648.

22 Chen WH, Guo YS, Zhang DH, Zhang HJ. Long-Term Prognosis of Suspected Myocarditis and Cardiomyopathy Associated with Viral Infection of the Myocardial Tissue: A Meta-Analysis of Cohort Studies. *Cardiovasc Ther* 2019; **2019**. doi:10.1155/2019/9342792.

23 Schultz JC, Hilliard AA, Cooper LT, Rihal CS. Diagnosis and Treatment of Viral Myocarditis. *Mayo Clin Proc* 2009; **84**: 1001–1009.

24 Chan KS, Zheng JP, Mok YW, Li YM, Liu YN, Chu CM *et al.* SARS: Prognosis, outcome and sequelae. Respirology. 2003; **8**. doi:10.1046/j.1440-1843.2003.00522.x.

25 Rogers JP, Chesney E, Oliver D, Pollak TA, McGuire P, Fusar-Poli P *et al.* Psychiatric and neuropsychiatric presentations associated with severe coronavirus infections: a systematic review and meta-analysis with comparison to the COVID-19 pandemic. *The Lancet Psychiatry* 2020; **0**. doi:10.1016/S2215-0366(20)30203-0.

26 Lee SH, Shin HS, Park HY, Kim JL, Lee JJ, Lee H *et al.* Depression as a mediator of chronic fatigue and post-traumatic stress symptoms in middle east respiratory syndrome survivors. *Psychiatry Investig* 2019; **16**: 59–64.

27 Hickie I, Davenport T, Wakefield D, Vollmer-Conna U, Cameron B, Vernon SD *et al.* Post-infective and chronic fatigue syndromes precipitated by viral and non-viral

For peer review only - http://bmjopen.bmj.com/site/about/guidelines.xhtml

pathogens: Prospective cohort study. *Br Med J* 2006; **333**: 575–578.

28 White PD. What Causes Prolonged Fatigue after Infectious Mononucleosis—and Does It Tell Us Anything about Chronic Fatigue Syndrome? *J Infect Dis* 2007; **196**: 4–5.

29 Aiyegbusi OL, Hughes SE, Turner G, Rivera SC, McMullan C, Chandan JS *et al.* Symptoms, complications and management of long COVID: a review. *J R Soc Med* 2021; **114**: 428–442.

30 Malik S, Asprusten TT, Pedersen M, Mangersnes J, Trondalen G, Van Roy B *et al.* Cognitive–behavioural therapy combined with music therapy for chronic fatigue following Epstein-Barr virus infection in adolescents: a randomised controlled trial. *BMJ Paediatr Open* 2020; **4**: e000797.

31 Bansal AS, Bradley AS, Bishop KN, Kiani-Alikhan S, Ford B. Chronic fatigue syndrome, the immune system and viral infection. Brain. Behav. Immun. 2012; **26**: 24–31.

32 Proal A, Marshall T. Myalgic encephalomyelitis/chronic fatigue syndrome in the era of the human microbiome: Persistent pathogens drive chronic symptoms by interfering with host metabolism, gene expression, and immunity. Front. Pediatr. 2018; **6**: 373.

33 Yamato M, Kataoka Y. Fatigue sensation following peripheral viral infection is triggered by neuroinflammation: Who will answer these questions? *Neural Regen Res* 2015; **10**: 203–204.

34 Islam MF, Cotler J, Jason LA. Post-viral fatigue and COVID-19: lessons from past epidemics. *Fatigue Biomed Heal Behav* 2020; **8**: 61–69.

35 Vernon SD, Whistler T, Cameron B, Hickie IB, Reeves WC, Lloyd A. Preliminary evidence of mitochondrial dysfunction associated with post-infective fatigue after acute infection with Epstein Barr Virus. *BMC Infect Dis* 2006; **6**: 15.

36 Holgate ST, Komaroff AL, Mangan D, Wessely S. Chronic fatigue syndrome: Understanding a complex illness. Nat. Rev. Neurosci. 2011; **12**: 539–544.

37 Crook H, Raza S, Nowell J, Young M, Edison P. Long covid—mechanisms, risk factors, and management. *BMJ* 2021; **374**: n1648.

38 UKRI. Outcomes of the UK COVID-19 Therapeutics Advisory Panel (UK-CTAP). 2021.https://www.ukri.org/about-us/policies-standards-and-data/data-collection/uk-covid-19-therapeutics-advisory-panel/ (accessed 27 Sep2021).

39 Page MJ, McKenzie JE, Bossuyt PM, Boutron I, Hoffmann TC, Mulrow CD *et al.* The PRISMA 2020 statement: an updated guideline for reporting systematic reviews. *BMJ* 2021; **372**. doi:10.1136/BMJ.N71.

40 Shamseer L, Moher D, Clarke M, Ghersi D, Liberati A, Petticrew M *et al.* Preferred reporting items for systematic review and meta-analysis protocols (prisma-p) 2015: Elaboration and explanation. BMJ. 2015; **349**. doi:10.1136/bmj.g7647.

41 Bennett JE, Dolin R, Blaser MJ. *Principles and practice of infectious diseases*. 2019.

42 World Health Organization. A clinical case definition of post COVID-19 condition by a Delphi consensus, 6 October 2021.

For peer review only - http://bmjopen.bmj.com/site/about/guidelines.xhtml

2021.https://www.who.int/publications/i/item/WHO-2019-nCoV-Post_COVID-19_condition-Clinical_case_definition-2021.1 (accessed 2 Mar2022).

43 Veritas Health Innovation. Covidence systematic review software. .

44 Shea BJ, Reeves BC, Wells G, Thuku M, Hamel C, Moran J *et al.* AMSTAR 2: a critical appraisal tool for systematic reviews that include randomised or non-randomised studies of healthcare interventions, or both. *BMJ* 2017; **358**: j4008.

45 Wells G, Shea B, O'Connell D, Peterson J, Welch V, Losos M *et al.* The Newcastle-Ottawa Scale (NOS) for assessing the quality of nonrandomised studies in meta-analyses. Secondary The Newcastle-Ottawa Scale (NOS) for assessing the quality of nonrandomised studies in meta-analyses. *Ottawa Hosp Res Inst* 2015; : 2–4.

46 Sterne JAC, Savović J, Page MJ, Elbers RG, Blencowe NS, Boutron I *et al.* RoB 2: A revised tool for assessing risk of bias in randomised trials. *BMJ* 2019; **366**. doi:10.1136/bmj.l4898.

47 Sterne JA, Hernán MA, Reeves BC, Savović J, Berkman ND, Viswanathan M *et al.* ROBINS-I: A tool for assessing risk of bias in non-randomised studies of interventions. *BMJ* 2016; **355**. doi:10.1136/bmj.i4919.

48 Cochrane. 16.1.2 General principles for dealing with missing data. https://handbook-5-1.cochrane.org/chapter_16/16_1_2_general_principles_for_dealing_with_missing_data.htm (accessed 10 Sep2021).

49 Guyatt GH, Oxman AD, Vist GE, Kunz R, Falck-Ytter Y, Alonso-Coello P *et al.* GRADE: An emerging consensus on rating quality of evidence and strength of recommendations. *BMJ* 2008. doi:10.1136/bmj.39489.470347.ad.

50 Staniszewska S, Brett J, Simera I, Seers K, Mockford C, Goodlad S *et al.* GRIPP2 reporting checklists: Tools to improve reporting of patient and public involvement in research. *BMJ* 2017; **358**: 3453.

For peer review only

For peer review only - http://bmjopen.bmj.com/site/about/guidelines.xhtml

Supplementary 1: PRISMA-P checklist

PRISMA-P (Preferred Reporting Items for Systematic review and Meta-Analysis Protocols) checklist: recommended items to address in a systematic review protocol*

Section and topic	Item No	Checklist item	Location in manuscript (page number)
**ADMINISTRATIVE INFORMATION**			
Title:			1
Identification	1a	Identify the report as a protocol of a systematic review	
Update	1b	If the protocol is for an update of a previous systematic review, identify as such	N/A
Registration	2	If registered, provide the name of the registry (such as PROSPERO) and registration number	6
Authors:			1
Contact	3a	Provide name, institutional affiliation, e-mail address of all protocol authors; provide physical mailing address of corresponding author	
Contributions	3b	Describe contributions of protocol authors and identify the guarantor of the review	1
Amendments	4	If the protocol represents an amendment of a previously completed or published protocol, identify as such and list changes; otherwise, state plan for documenting important protocol amendments	N/A
Support:			

For peer review only

Sources	5a	Indicate sources of financial or other support for the review	1
Sponsor	5b	Provide name for the review funder and/or sponsor	1
Role of sponsor or funder	5c	Describe roles of funder(s), sponsor(s), and/or institution(s), if any, in developing the protocol	1
**INTRODUCTION**			
Rationale	6	Describe the rationale for the review in the context of what is already known	5
Objectives	7	Provide an explicit statement of the question(s) the review will address with reference to participants, interventions, comparators, and outcomes (PICO)	6
**METHODS**			
Eligibility criteria	8	Specify the study characteristics (such as PICO, study design, setting, time frame) and report characteristics (such as years considered, language, publication status) to be used as criteria for eligibility for the review	7
Information sources	9	Describe all intended information sources (such as electronic databases, contact with study authors, trial registers or other grey literature sources) with planned dates of coverage	8
Search strategy	10	Present draft of search strategy to be used for at least one electronic database, including planned limits, such that it could be repeated	8 and supplementary table 2
Study records:			
Data management	11a	Describe the mechanism(s) that will be used to manage records and data throughout the review	9

For peer review only

Selection process	11b	State the process that will be used for selecting studies (such as two independent reviewers) through each phase of the review (that is, screening, eligibility and inclusion in meta-analysis)	9
Data collection process	11c	Describe planned method of extracting data from reports (such as piloting forms, done independently, in duplicate), any processes for obtaining and confirming data from investigators	9 and supplementary table 3
Data items	12	List and define all variables for which data will be sought (such as PICO items, funding sources), any pre-planned data assumptions and simplifications	9
Outcomes and prioritization	13	List and define all outcomes for which data will be sought, including prioritization of main and additional outcomes, with rationale	9
Risk of bias in individual studies	14	Describe anticipated methods for assessing risk of bias of individual studies, including whether this will be done at the outcome or study level, or both; state how this information will be used in data synthesis	9
Data synthesis	15a	Describe criteria under which study data will be quantitatively synthesised	10
	15b	If data are appropriate for quantitative synthesis, describe planned summary measures, methods of handling data and methods of combining data from studies, including any planned exploration of consistency (such as I^2, Kendall's τ)	10
	15c	Describe any proposed additional analyses (such as sensitivity or subgroup analyses, meta-regression)	10
	15d	If quantitative synthesis is not appropriate, describe the type of summary planned	10

For peer review only

Meta-bias(es)	16	Specify any planned assessment of meta-bias(es) (such as publication bias across studies, selective reporting within studies)	10
Confidence in cumulative evidence	17	Describe how the strength of the body of evidence will be assessed (such as GRADE)	11

*** It is strongly recommended that this checklist be read in conjunction with the PRISMA-P Explanation and Elaboration (cite when available) for important clarification on the items. Amendments to a review protocol should be tracked and dated. The copyright for PRISMA-P (including checklist) is held by the PRISMA-P Group and is distributed under a Creative Commons Attribution Licence 4.0.**

For peer review only

Supplementary 2: Draft search terms

Search terms

Virus terms

- Middle eastern respiratory syndrome
    - MERS
- Severe acute respiratory syndrome
    - SARS
- Coronavirus
- Ebola
- Marburg
- Epstein Barr virus
- Cytomegalovirus
- Varicella Zoster Virus
- Other Human Herpes Viruses
- Herpes Simplex Virus
- Influenza
- Influenza A
- Influenza B
- Influenza C
- Influenza D
- Coxsackie
- Hepatitis A
- Enterovirus
- Rhinovirus
- Parvovirus
- Ross River Virus
- Chikungunya
- Semliki Forest Complex Viruses
- West Nile River virus
- Dengue
- Yellow Fever
- Zika
- Rotavirus
- Mumps
- Measles
- Parainfluenza
- Respiratory Syncytial Virus
- Human Metapneumovirus
- Adenoviruses
- Norovirus

For peer review only

- Sapovirus
- Astrovirus*
- COVID

Long-term phrases

- Long-term
- Longterm
- Persistent
- Recurrent
- Chronic
- Post-viral
- Post-infectious
- Post infectious

Treatment terms

- Treatment*
- Therap*
- Intervention

For peer review only

Supplementary 3: Draft data extraction table

Author (year)	Setting	Participant characteristics				Name of virus	Outcomes	Intervention	Comparator	Main findings
		N	Mean age, n (SD)	Sex	Ethnicity (%)					

For peer review only
